# Improving local prevalence estimates of SARS-CoV-2 infections using a causal debiasing framework

**DOI:** 10.1038/s41564-021-01029-0

**Published:** 2021-12-31

**Authors:** George Nicholson, Brieuc Lehmann, Tullia Padellini, Koen B. Pouwels, Radka Jersakova, James Lomax, Ruairidh E. King, Ann-Marie Mallon, Peter J. Diggle, Sylvia Richardson, Marta Blangiardo, Chris Holmes

**Affiliations:** 1 University of Oxford, Oxford, UK; 2 The Alan Turing Institute and Royal Statistical Society Statistical Modelling and Machine Learning Laboratory, London, UK; 3 MRC Centre for Environment and Health, Department of Epidemiology and Biostatistics, Imperial College London, London, UK; 4 Health Economics Research Centre, Nuffield Department of Population Health, University of Oxford, Oxford, UK; 5 The National Institute for Health Research Health Protection Research Unit in Healthcare Associated Infections and Antimicrobial Resistance at the University of Oxford, University of Oxford, Oxford, UK; 6 The Alan Turing Institute, London, UK; 7 MRC Harwell Institute, Harwell, UK; 8 CHICAS, Lancaster Medical School, Lancaster University, Lancaster, UK; 9 MRC Biostatistics Unit, University of Cambridge, Cambridge, UK

**Keywords:** Epidemiology, Viral infection

## Abstract

Global and national surveillance of SARS-CoV-2 epidemiology is mostly based on targeted schemes focused on testing individuals with symptoms. These tested groups are often unrepresentative of the wider population and exhibit test positivity rates that are biased upwards compared with the true population prevalence. Such data are routinely used to infer infection prevalence and the effective reproduction number, *R*_*t*_, which affects public health policy. Here, we describe a causal framework that provides debiased fine-scale spatiotemporal estimates by combining targeted test counts with data from a randomized surveillance study in the United Kingdom called REACT. Our probabilistic model includes a bias parameter that captures the increased probability of an infected individual being tested, relative to a non-infected individual, and transforms observed test counts to debiased estimates of the true underlying local prevalence and *R*_*t*_. We validated our approach on held-out REACT data over a 7-month period. Furthermore, our local estimates of *R*_*t*_ are indicative of 1-week- and 2-week-ahead changes in SARS-CoV-2-positive case numbers. We also observed increases in estimated local prevalence and *R*_*t*_ that reflect the spread of the Alpha and Delta variants. Our results illustrate how randomized surveys can augment targeted testing to improve statistical accuracy in monitoring the spread of emerging and ongoing infectious disease.

## Abstract (web summary)

A causal debiasing framework provides accurate estimates of local prevalence and effective reproduction number for surveillance of SARS-CoV-2 cases using data from randomized testing schemes to model ascertainment bias in targeted subpopulation data.

## Main

The spread of the new severe acute respiratory syndrome coronavirus 2 (SARS-CoV-2) and the ensuing outbreaks of coronavirus disease 2019 (COVID-19) have placed a substantial burden on public health in the United Kingdom. As of 14 July 2021, the number of people recorded to have died in the United Kingdom within 28 days of a positive SARS-CoV-2 test was 128,530 (refs. ^1,2^). In response to the ongoing epidemic, the UK government has implemented a number of non-pharmaceutical interventions to reduce the transmission of SARS-CoV-2, ranging from localized measures, such as the closures of bars and restaurants, to full national lockdowns^3^. The localized measures have been employed through a regional tier system, with lower tier local authorities (LTLAs) being placed under varying levels of restrictions according to data such as the number of positive polymerase chain reaction (PCR) tests returned there over a 7-day interval (or local weekly positive tests)^4^. Following a third national lockdown that began on the 6 January 2021, the United Kingdom has undergone a staged relaxation of restrictions, with lockdown rules ending on 19 July 2021 (ref. ^5^).

In the United Kingdom, there are two major ongoing studies that undertake randomized survey testing to provide an insight into the prevalence of SARS-CoV-2. Since April 2020, the Office for National Statistics (ONS) COVID-19 Infection Survey (CIS) tests a random sample of people living in the community with longitudinal follow-up^6^. The survey is designed to be representative of the UK population, with individuals aged two years and over in private households randomly selected from address lists and previous ONS surveys, although it does not explicitly cover care homes, the sheltering population, student halls or individuals currently being hospitalized. The REal-time Assessment of Community Transmission (REACT) study is a second nationally representative prevalence survey of SARS-CoV-2 based on repeated cross-sectional samples from a representative subpopulation defined via (stratified) random sampling from the National Health Service patient register of England^7,8^. Importantly, both surveys recruit participants regardless of symptom status and are therefore able to largely avoid issues arising from ascertainment bias when estimating prevalence. The ONS CIS uses multilevel regression and post-stratification to account for any residual ascertainment effects due to non-response^6^, whereas the REACT study uses survey weights for this purpose.

While randomized surveillance testing readily provides an accurate statistical estimate of prevalence of PCR positivity, precision can be low at finer spatiotemporal scales (for example, at the LTLA level), even in large studies such as the ONS CIS and REACT surveys. Our major goal here is to unlock the information in non-randomized testing under arbitrary, unknown ascertainment bias. Although we expect the methods to apply in a broad manner, here we focus on Pillar 1 and Pillar 2 (Pillar 1+2) PCR tests conducted in England between 31 May 2020 and 20 June 2021 (lateral flow device (LFD) tests are not included; further details provided in Methods and Data availability). Pillar 1 tests refer to “all swab tests performed in Public Health England (PHE) labs and National Health Service (NHS) hospitals for those with a clinical need, and health and care workers”^9^, and Pillar 2 tests comprise “swab testing for the wider population”^9^. Pillar 1+2 testing therefore has more capacity than the randomized programmes, but the protocol incurs ascertainment bias because those at increased risk of being infected are tested, such as frontline workers, contacts traced to a COVID-19 case or the subpopulation presenting with COVID-19 symptoms, such as loss of taste and smell^9^. Hence, raw prevalence estimates from Pillar 1+2 data (as a proportion of tested population) will tend to be biased upwards and cannot directly be used to estimate the unknown infection rate in a region. In contrast, as a proportion of the entire population, the bias is downwards as not all individuals with infection in the area are captured. Furthermore, the degree of upward bias may be influenced by overall testing capacity and uptake. In addition, the raw prevalence estimates tend not to capture asymptomatic infection, even though there is evidence to indicate that asymptomatic individuals can contribute to viral transmission^10,11^.

Combining data from multiple surveillance schemes can improve estimates for prevalence. For example, Manzi et al.^12^ incorporated information from multiple, biased, commercial surveys to provide more accurate and precise estimates of smoking prevalence in local authorities across the East of England. A number of geostatistical frameworks for infectious disease modelling based on multiple diagnostic tests have been developed^13–15^. These accommodate different sources of heterogeneity among the tests to deliver more reliable and precise inferences on disease prevalence.

**Fig. 1:**
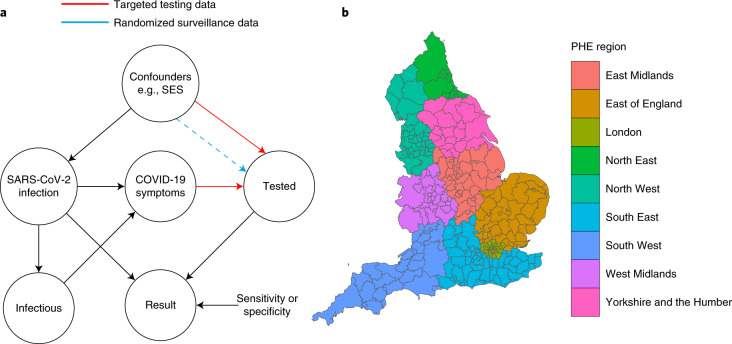
Causal diagram and spatial structure underlying the test count data. **a**, A DAG representing the causal models underlying SARS-CoV-2 swab testing data for targeted test-and-trace data (Pillar 1+2) and randomized surveillance data (for example, REACT). Randomization breaks the causal link between COVID-19 symptoms and swab testing. The nodes represent binary (yes/no) states for an individual in the relevant population. SES is shown as an example confounder (in addition to symptom status). The dashed line represents residual ascertainment effects stemming from non-ignorable non-response in the REACT study. **b**, A map of LTLAs in England and their corresponding PHE regions.

To understand the ascertainment bias problem and to enable a statistical approach to correction, it is helpful to consider a simplified causal model^16,17^ for Pillar 1+2 data. This is represented by a directed acyclic graph (DAG), shown in Fig. 1a, that charts the dependencies of an individual from infection status to test result. The circles indicate the binary (yes/no) states of an individual. The DAG characterizes the joint distribution of the major factors leading to the observed data. Throughout the paper, we use the term ‘targeted testing data’ to refer to data gathered under some ascertainment process distinct from (stratified) random sampling, with an exemplar being selection for testing of the subpopulation with COVID-19 symptoms, which comprises a sizeable proportion of Pillar 1+2 tests. There are several other potential confounders, exemplified in Fig. 1a by socioeconomic status (SES), which is a well-studied factor of both infection risk and access to healthcare and/or testing. The DAG explicitly characterizes statistically why we cannot directly use Pillar 1+2 data. The DAG also points to a potential solution that we pursue here: if the statistical dependencies as indicated by the arrows in Fig. 1a can be modelled, then we can correct for the ascertainment bias in Pillar 1+2 data.

In addition to prevalence, there are a number of epidemiological parameters that may be useful for informing localized non-pharmaceutical interventions. For example, one particular variable of interest is the (time-varying) effective reproductive number *R*_*t*_, which is defined roughly as the average number of infections caused by an infectious individual. That is, when *R*_*t*_ > 1, the epidemic will continue to spread. The current pandemic has spurred the development of models that aim to incorporate multiple sources of data to estimate important epidemiological parameters. See Supplementary Table 1 for an overview of the methodological work most related to ours^18–25^ (https://localcovid.info/), including a brief description of each method and what the data inputs and results outputted are; we also recommend refs. ^26,27^ for reviews, which have a particular focus on *R*_*t*_.

Within this urgent and fast developing area of research, it is clearly important to define the aspects in which our method contributes. First, we have developed methods to infer unbiased local prevalence, *I*_*t*_, from targeted testing data. This is important in its own right because being able to estimate local prevalence accurately from targeted testing data adds an important facet to existing COVID-19 monitoring capabilities. Here, we focus on weekly period prevalence and explicitly target the number of infectious individuals via a correction to the estimated PCR-positive numbers. Second, our method outputs bias-adjusted cross-sectional prevalence likelihoods *p*(*n*_*t*_ of *N*_*t*_ ∣ *I*_*t*_), where *n*_*t*_ and *N*_*t*_ are positive and total targeted test counts, respectively. This allows prevalence information from targeted data to be coherently embedded in a modular way into complex spatiotemporal epidemiological models, including those synthesizing multiple data types. We exemplify this by implementing a susceptible-infectious-recovered (SIR) model around our ascertainment model likelihood. Third, our local ascertainment model is based on targeted testing data alone with both the number of positive and total tests being modelled (*n*_*t*_ and *N*_*t*_). This has two important benefits: spatiotemporal variation in testing uptake and capacity is explicitly conditioned on (via *N*_*t*_), and differential test specificity and sensitivity can be naturally incorporated into our causal ascertainment model.

## Results

### Correcting for ascertainment bias in targeted testing data

**Fig. 2:**
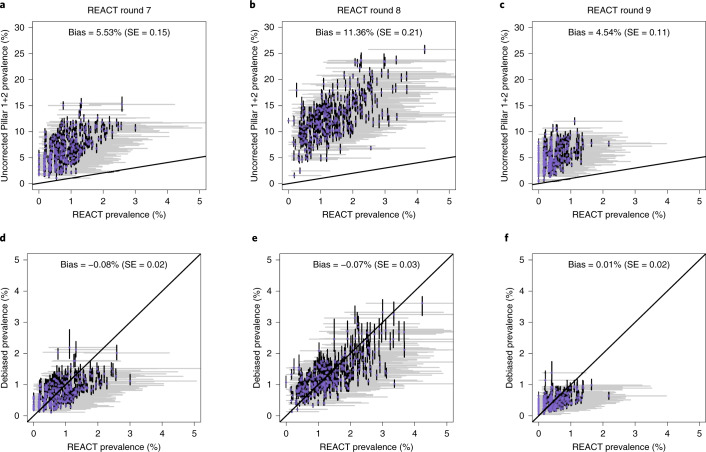
Uncorrected (top) and corrected (bottom) Pillar 1+2 prevalence estimates against REACT estimates. **a**–**f**, Uncorrected (raw positivity rates) and corrected (debiased) Pillar 1+2 PCR-positive prevalence estimates against (gold-standard) REACT estimates from randomized surveillance. Each point corresponds to a LTLA. Each scatter plot compares pillar 1+2 prevalence estimates against unbiased estimates from the REACT study. **a**,**d**, REACT round 7 data (13 November 2020 to 3 December 2020). **b**,**e**, Round 8 (6–22 January 2021. **c**,**f**, Round 9 (4–23 February 2021). Uncorrected results are shown in **a**–**c** and bias-corrected cross-sectional estimates in **d**–**f**. Horizontal grey lines are 95% exact binomial confidence intervals from the REACT data. The number of independent tests underlying each mean and (horizontal) credible intervals for the REACT data varied between 248 and 2,387. Vertical black lines in **a**–**c** are 95% exact binomial confidence intervals for from the raw, non-debiased Pillar 1+2 data. Vertical black lines in **d**–**f** are 95% posterior credible intervals from the debiased Pillar 1+2 data. The number of independent tests underlying each mean and (vertical) credible interval for the Pillar 1+2 data varied between 1,117 and 42,458. Neither set of prevalence estimates has been corrected for false positives or negatives. Note that in **d**–**f**, the credible interval widths are systematically tighter for the debiased Pillar 1+2 compared with the REACT data, which highlights the useful information content in debiased Pillar 1+2 data.

Figure 2a–c displays the percentage of positive Pillar 1+2 tests (as a proportion of those tested) against accurate prevalence estimates from the REACT study, which shows a clear upward bias (each point corresponds to a single LTLA). Here, we introduce a bias-correction method that aims to provide accurate estimates of prevalence at the local level, as displayed in Fig. 2d–f, based on the posterior cross-sectional prevalence *p*(*I*_*t*_ ∣ *n*_*t*_ of *N*_*t*_).

With reference to the causal DAG in Fig. 1a, we define the essential bias parameter, *δ*, as

1 $$\delta :={{\mathrm{log}}}\,\left(\frac{{{{\rm{odds}}}}(\,{{\mbox{tested}}}\,| \,{{\mbox{infected}}}\,)}{{{{\rm{odds}}}}(\,{{\mbox{tested}}}\,|\, {{\mbox{not}}}\,\,\,{{\mbox{infected}}}\,)}\right)$$

that is, the log odds-ratio of being tested in the infected subpopulation versus in the non-infected subpopulation. Larger values of *δ* generally correspond to higher levels of ascertainment bias; that is, a higher chance of an individual with an infection being selected for testing relative to an individual without infection.

**Fig. 3:**
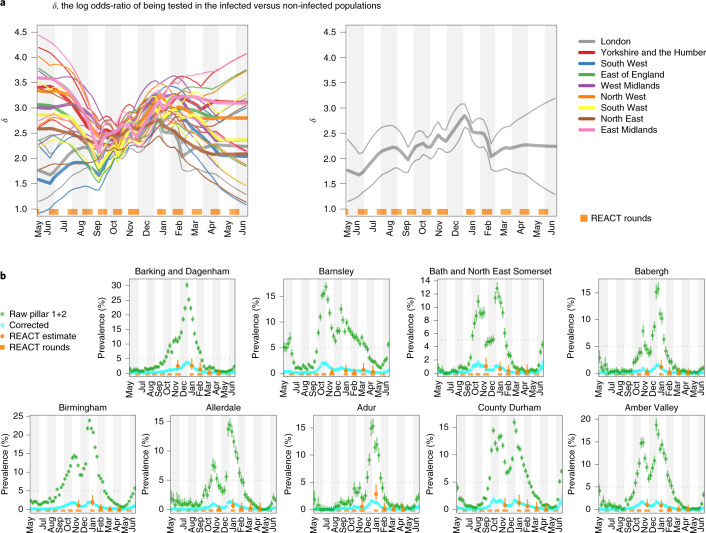
Ascertainment bias parameters and LTLA-level prevalence estimates. **a**, Smooth EB priors on bias parameters *δ*_1:*T*_. Left: heterogeneous bias across the nine PHE regions. Right: London only. The thick curves show the prior means and the narrow curves show 95% credible intervals. Note that *δ* is the log odds-ratio, so, for example, *δ* = 3 implies that the odds of being tested are *e*^3^ ≈ 20 times higher in individuals with infection compared with individuals without infection. **b**, LTLA-level prevalence estimates: raw Pillar 1+2 estimates (that is, positivity rate), cross-sectionally corrected Pillar 1+2 and gold-standard REACT estimates. For each of the nine PHE regions, we present the constituent LTLA whose name is ranked top alphabetically. The number of independent tests underlying each (orange) mean and credible interval based on the REACT data varied between 288 and 620. The number of independent tests underlying each (green or cyan) mean and credible interval based on the Pillar 1+2 data varied between 390 and 43,650. The green symbols and error bars show the mean exact binomial 95% confidence intervals. The cyan symbols and error bars show posterior median and 95% credible intervals. The orange symbols and error bars show the mean and 95% exact binomial confidence intervals.

Our approach combines randomized surveillance data (REACT) and targeted surveillance data (Pillars 1+2) to infer *δ* at the coarse geographical level (PHE region; Fig. 1b). We then take forward this information by specifying a temporally smooth empirical Bayes (EB) prior on *δ*_1:*T*_, applied to each constituent local region (LTLA) in the local prevalence analyses. Figure 3a shows the resulting EB priors on *δ*. There is potentially more variation in *δ* across regions early and late in the sampling period (before September 2020 and after March 2021), although the prior credible intervals are broad and often overlapping. The data provide more information on *δ* between October 2020 and February 2021.

### Cross-sectional local prevalence from targeted testing data

#### Debiased likelihood for modular sharing of prevalence information

Equipped with a coarse-scale (PHE-region level) EB prior on bias *δ*, we evaluated a fine-scale (LTLA-level) *δ*-marginalized likelihood of the form $p({n}_{\mathit{t}}\,{{\mbox{of}}}\,{N}_{\mathit{t}}| {I}_{\mathit{t}},{\hat{\nu }}_{\mathit{t}})$ as described in equation (17) in the Methods (“Cross-sectional inference on local prevalence”). This debiased prevalence likelihood can be readily exported and modularly incorporated into more complex models, as we illustrate below (“Longitudinal local prevalence and transmission”).

#### Cross-sectional prevalence posterior

The *δ*-marginalized likelihood can be inputted directly into cross-sectional Bayesian inference, outputting the prevalence posterior $p({I}_{t}| {n}_{t}\,{{\mbox{of}}}\,{N}_{t},{\hat{\nu }}_{t})$ for each time point at which such count data are available. Figure 3b plots these cross-sectional prevalence posteriors beneath the raw counts for a subset of LTLAs across the nine PHE regions. REACT sampling periods are plotted at the base of each panel, and local prevalence estimates from REACT round 7 (November 2020) and round 8 (January 2021) are also superimposed. The corrected cross-sectional prevalence estimates are consistent with the gold-standard REACT estimates, but are more precise, as expected from Bayesian principles of data synthesis.

### Longitudinal local prevalence and transmission

**Fig. 4:**
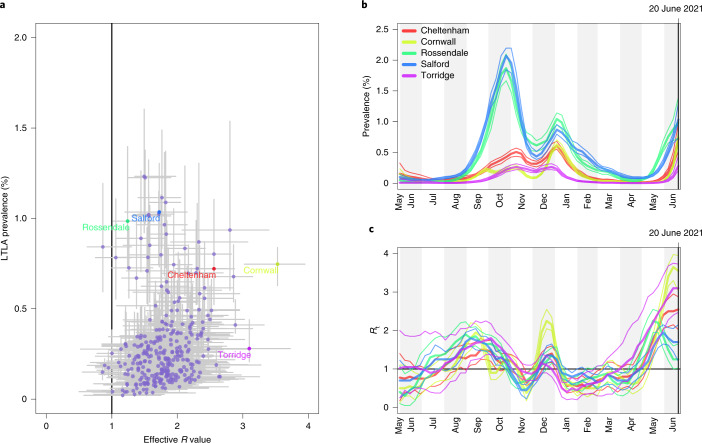
Outputs of the longitudinal local prevalence model. **a**, Scatterplot of prevalence against effective *R* number (each point corresponds to one LTLA) for the week of 20 June 2021. **b**, Longitudinal posteriors for prevalence at a selection of LTLAs. **c**, Longitudinal posteriors for *R*_*t*_ at a selection of LTLAs. The vertical line and horizontal line in **b** and **c**, respectively, indicate an effective reproduction number of *R*_*t*_ = 1; when *R*_*t*_ > 1, the number of cases occurring in a population will increase. In **a**, the symbols show posterior medians and the error bars show 95% credible intervals. In **b** and **c**, the thick lines show posterior medians and the narrow lines show 95% credible intervals.

**Extended Data Fig. 1:**
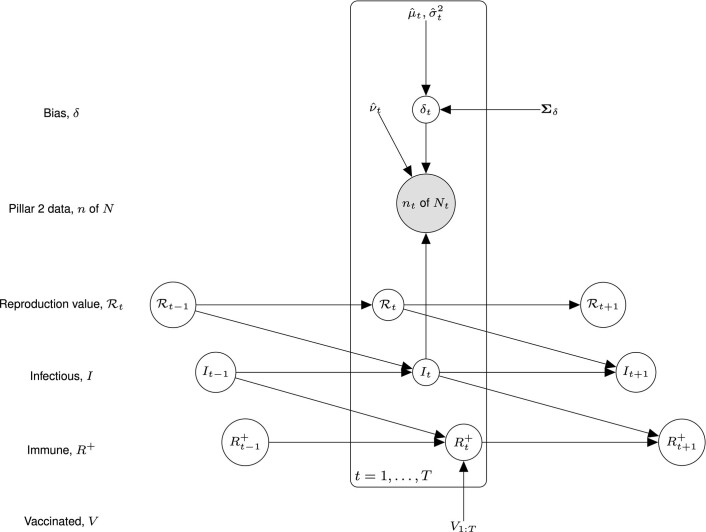
Longitudinal model DAG for SIR epidemic model at local level (for example LTLA). Directed paths characterise conditional probability distributions, in contrast to the paths showing transitions between model compartments in Supplementary Fig. 1. Inference is for a region, for example an LTLA, based only on targeted test data collected in this region, *n*_*t*_ of *N*_*t*_. A prior on *δ*_*t*_ parameterized (${\hat{\mu }}_{t}$, ${\hat{\sigma }}_{t}^{2}$) brings information on the Pillar 1+2 ascertainment bias learned from randomized surveillance testing data available for the PHE region in which the LTLA lies. The *T* × *T* covariance matrix ***Σ***_*δ*_ imparts temporal smoothness on *δ*_1:*T*_. Effective reproduction numbers are denoted ${{{{\mathcal{R}}}}}_{1:T}$, number of infectious individuals by *I*_1:*T*_, and the number of immune individuals by ${R}_{1:T}^{+}$.

The cross-sectional debiased likelihood can be introduced modularly into a wide variety of downstream epidemiological models. We illustrate this by using the likelihood as an input to a simple SIR epidemic model (Methods, “Full Bayesian inference under a stochastic SIR epidemic model”, and Extended Data Fig. 1). Figure 4a plots the estimated prevalence against *R*_*t*_ number at the most recent time point (the week of 20 June 2021), with each point corresponding to a single LTLA. The scatter plot provides a quick visual representation of regions where transmission rates and/or prevalence are relatively high. To illustrate, we label five LTLAs with high prevalence and/or *R*_*t*_ estimates. The estimated longitudinal prevalence and *R*_*t*_ for this subset of LTLAs (Fig. 4b,c) can help further characterize the longitudinal dynamics of prevalence and transmission in the time interval leading up to 20 June 2021. In particular, the data show the estimated rate of change in prevalence and separately indicate whether *R*_*t*_ is increasing or decreasing.

**Fig. 5:**
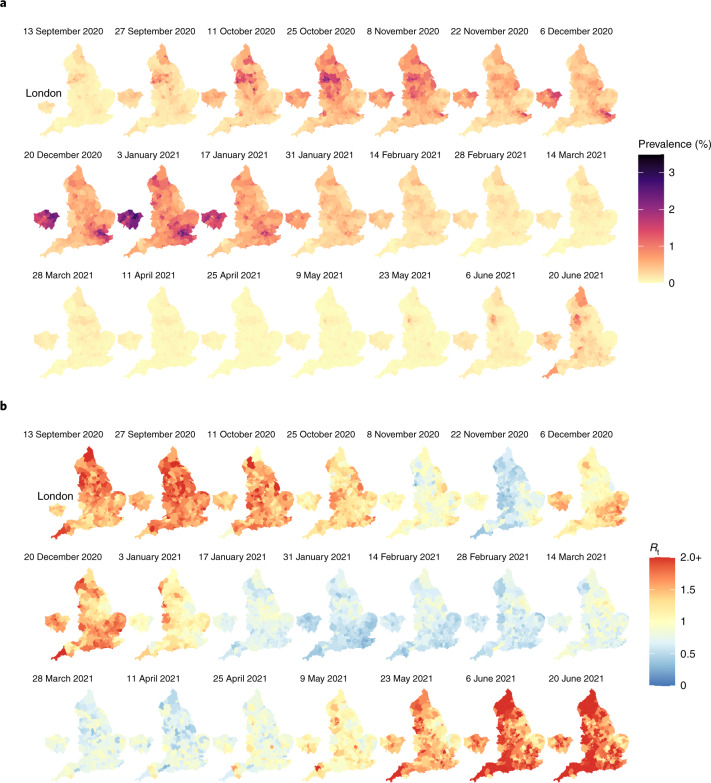
Maps of estimated prevalence and effective reproduction number. **a**, Fortnightly maps of estimated local prevalence in England from 13 September 2020 to 20 June 2021. **b**, Fortnightly maps of estimated local *R*_*t*_ in England from 13 September 2020 to 20 June 2021.

Figure 5a displays the spatiotemporal local prevalence and Fig. 5b displays *R*_*t*_, using a fortnightly sequence of maps, with each LTLA coloured according to its estimate prevalence or *R*_*t*_. Zoom-in boxes display the local fine-scale structure for London.

### Relating local prevalence and transmission to spread of the variants of concern

A striking feature of the maps in Fig. 5a is the increasing prevalence in London throughout November to December 2020. This is consistent with the known arrival of the Alpha variant of concern (VoC) 202012/01 (lineage B.1.1.7) that emerged in the South East of England in November 2020, and has been estimated to have a 43–90% higher reproduction number than pre-existing variants^28^. Similarly, the increase in *R*_*t*_ from May 2021 onwards is in accordance with the spread of the Delta VoC 21APR-02 (lineage B.1.617.2), which is estimated to have a reproduction number approximately 60% higher than that of the Alpha VoC^29^.

**Fig. 6:**
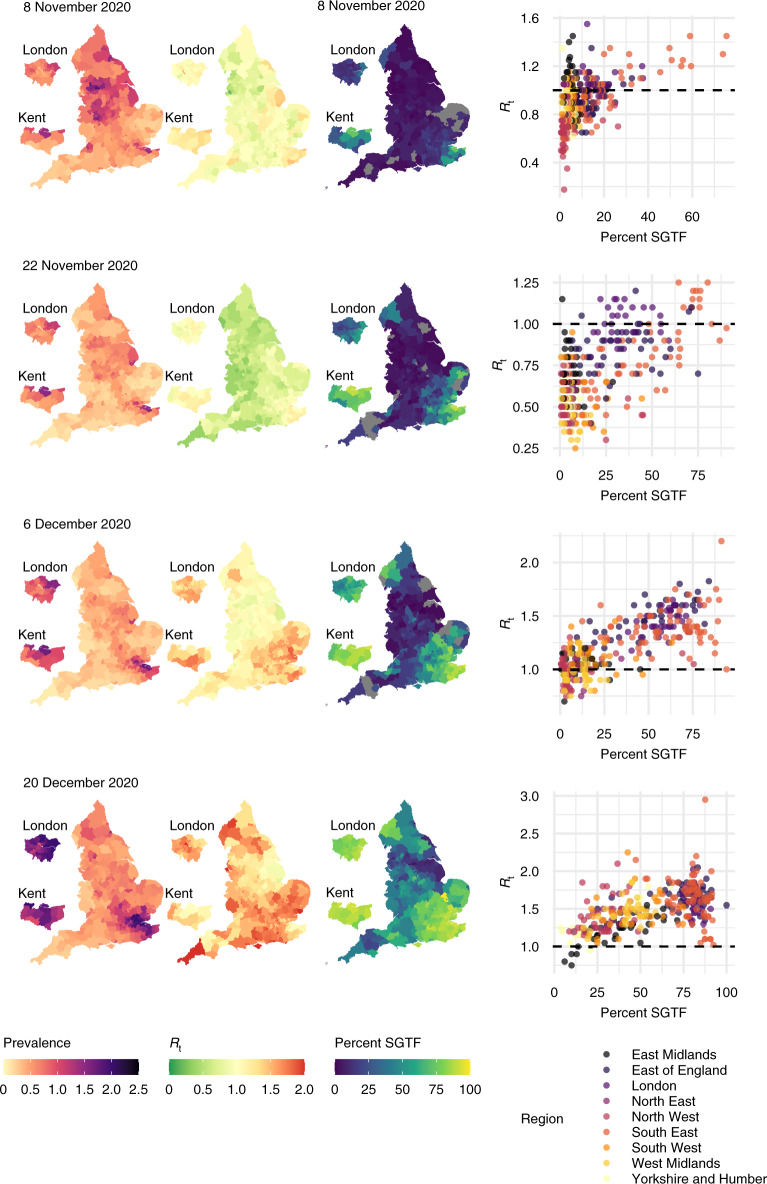
Maps of estimated prevalence, effective reproduction number and Alpha variant frequency. Maps of estimated local prevalence (left), estimated local *R*_*t*_ (middle) and frequency of SGTF (right), and scatter plots of SGTF frequency against estimated *R*_*t*_ (far right). Grey-coloured LTLAs denote missing data.

Similar to a previous study^28^, we characterized the relationship between the estimated local *R*_*t*_ and the frequency of Alpha VoC 202012/01, as approximated by the frequency of *S* gene target failure (SGTF) in Taqpath sequencing assays used during this time period^30^. Figure 6 illustrates the spatial distributions of the Alpha VoC 202012/01 against estimated prevalence and estimated *R*_*t*_ from mid-November 2020 to mid-December 2020. The increase in frequency of the VoC was initially isolated to the South East but then spread outwards, accompanied by a corresponding increase in both local estimated prevalence and *R*_*t*_. We observe a strong positive association between the local VoC frequency and estimated local *R*_*t*_, which are consistent with the increased transmissibility of this VoC identified in ref. ^28^.

**Extended Data Fig. 2:**
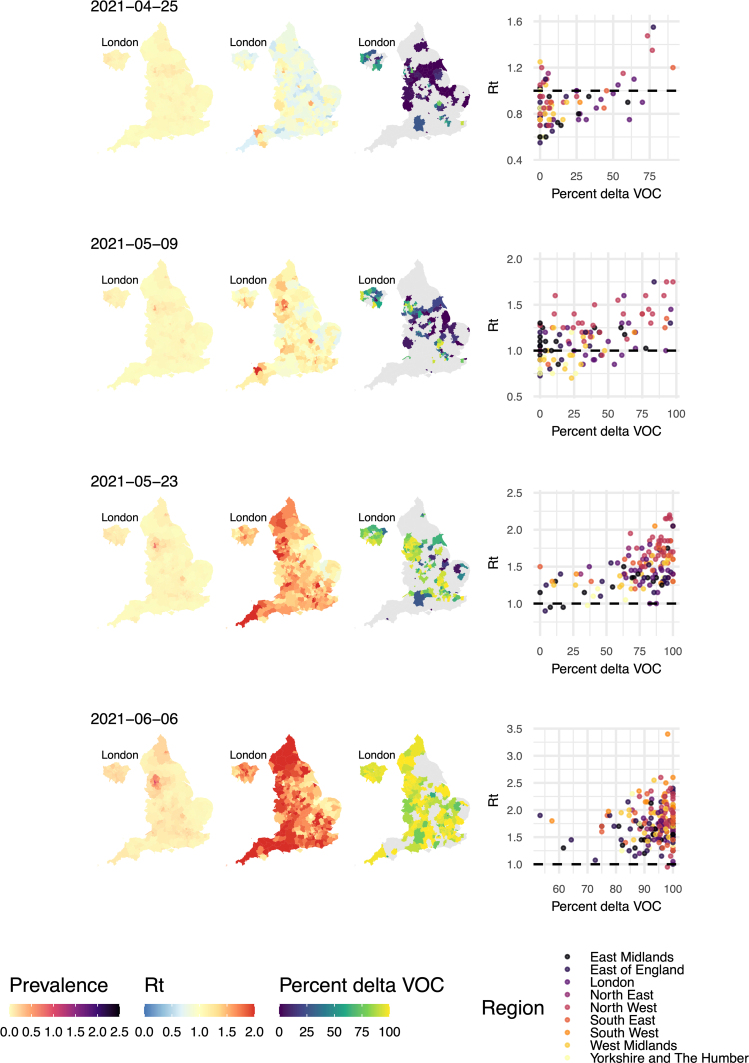
Maps of estimated local prevalence (left), estimated local *R*_*t*_ (middle), and frequency of the delta variant (right), and scatter plot of Delta variant frequency against estimated *R*_*t*_. Grey-coloured areas denote where the total number of variant sequencing assays performed (across all variants) is less than 10; in these cases the delta variant frequency estimates are omitted due to having high standard error.

We performed a similar analysis for the Delta VoC 21APR-02 using data provided by the Wellcome Sanger Institute’s Covid-19 Genomics Initiative^31^. Extended Data Fig. 2 shows the spatial distributions of the Delta VoC 21APR-02 against estimated prevalence and estimated *R*_*t*_ from the end of April 2021 to the start of June 2021. We see that the Delta VoC becomes the dominant variant over the course of this time period, and in contrast to the Alpha VoC, the spread of the variant was not isolated to a single region of England. We again observe a strong positive association between the local VoC frequency and estimated local *R*_*t*_. A simple linear regression of *R*_*t*_ against Delta frequency for the week of 23 May 2021 indicated an increase in transmissibility of 0.55 (0.39–0.71) due to the Delta VoC, which is in accordance with estimates obtained in ref. ^29^.

### Accuracy validation using ultra-coarse and incomplete data to estimate *δ*

**Extended Data Fig. 3:**
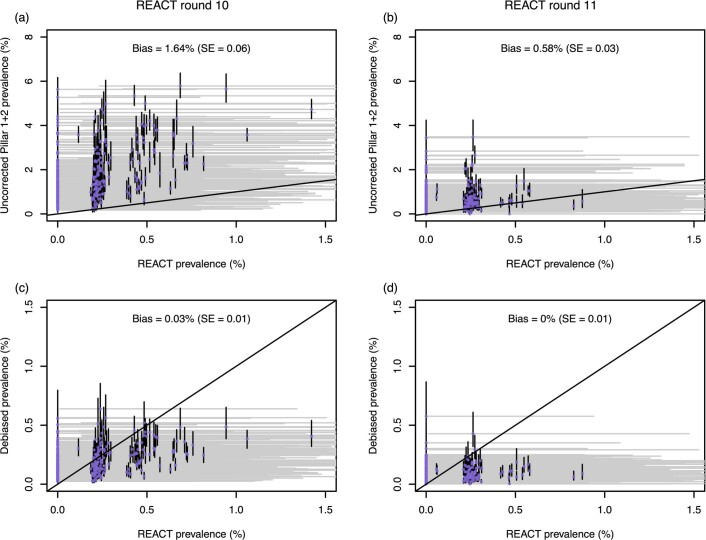
Uncorrected (raw positivity rates) and corrected (debiased) Pillar 1+2 PCR-positive prevalence estimates against (gold-standard) REACT estimates from randomised surveillance for REACT rounds 10 and 11. Each point corresponds to an LTLA. Each scatter plot compares Pillar 1+2 prevalence estimates against unbiased estimates from the REACT study. Panels (a,c) show REACT round 10 data (11th Mar - 30th Mar 2021), and panels (b,d) show round 11 (15th Apr - 3rd May 2021). Uncorrected results are shown in panels (a-b) and bias-corrected cross-sectional estimates in (c-d). Horizontal grey lines are 95% exact binomial confidence intervals from the REACT data. Vertical black lines in panels (a-b) are 95% exact binomial confidence intervals from the raw, non-debiased Pillar 1+2 data. Vertical black lines in panels (c-d) are 95% posterior credible intervals from the debiased Pillar 1+2 data. Neither set of prevalence estimates has been corrected for false positives/negatives. Note that in panels (c-d), the CI widths are systematically tighter for the debiased Pillar 1+2 compared to the REACT data, pointing to the useful information content in debiased Pillar 1+2 data. The number of independent tests underlying each mean and (horizontal) CI for the REACT data varied between 289 and 1,894. The number of independent tests underlying each mean and (vertical) CI for the Pillar 1+2 data varied between 977 and 29,998.

We assessed the performance of debiased fine-scale (LTLA-level) prevalence estimates by measuring how well they predict LTLA-level REACT data. The validation is best described in terms of coarse-scale REACT training data and contemporaneous fine-scale REACT test data. The training data inputted are REACT PHE-region-level and Pillar 1+2 LTLA-level positive (and number of) test counts for the week at the centre of the corresponding REACT round to be predicted. The test data are REACT LTLA-level positive (and number of) test counts aggregated across the relevant REACT sampling round. Figure 2 visually compares cross-sectional LTLA prevalence estimates from debiased targeted data (that is, based only on the training data) with accurate gold-standard estimates from REACT LTLA-level test data. The average estimated bias is reduced to low levels for comparisons with REACT round 7 (–0.08%, standard error (SE) = 0.02), round 8 (–0.07%, SE = 0.03) and round 9 (0.01%, SE = 0.02). Extended Data Fig. 3c,d displays analogous results for REACT rounds 10 and 11, with average estimated bias reduce to 0.03% (SE = 0.01) and 0% (SE = 0.01), respectively.

**Extended Data Fig. 4:**
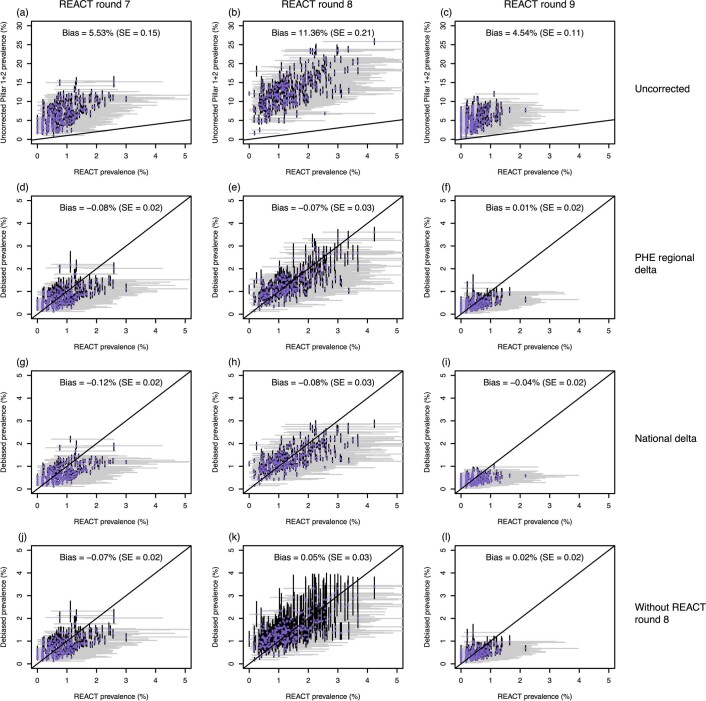
Uncorrected (raw positivity rates) and corrected (debiased) Pillar 1+2 PCR-positive prevalence estimates against (gold-standard) REACT estimates from *limited* randomised surveillance. Each point corresponds to an LTLA. Each scatter plot compares Pillar 1+2 prevalence estimates against unbiased estimates from the REACT study. Left to right the columns of panels show results from REACT round 7 (13th Nov - 3rd Dec 2020), round 8 (6th-22nd Jan 2021), and round 9 (4th-23rd Feb 2021). On the vertical axes: (a-c) show uncorrected test positivity rates; (d-f) show bias-corrected prevalence estimates; (g-i) show bias-corrected prevalence estimates where the bias *δ* was estimated at the ultra-coarse national level; and (j-l) show bias-corrected prevalence estimates where data from REACT round 8 was omitted, in order to assess the impact of a more limited randomised surveillance regime. Horizontal grey lines are 95% exact binomial confidence intervals from the REACT data. Vertical black lines in (a-c) are 95% exact binomial confidence intervals from the raw, non-debiased Pillar 1+2 data. Vertical black lines in panels (d-l) are 95% posterior credible intervals from the debiased Pillar 1+2 data. Neither set of prevalence estimates has been corrected for false positives/negatives. The number of independent tests underlying each mean and (horizontal) CI for the REACT data varied between 248 and 2,387. The number of independent tests underlying each mean and (vertical) CI for the Pillar 1+2 data varied between 1,117 and 42,458.

REACT and ONS CIS are among the most comprehensive randomized surveillance studies in the world. We have tried to assess how well the debiasing model might hold when we are faced with coarser-scale or more limited randomized testing data. First, to investigate the downstream effects of ultra-coarse-scale randomized surveillance data, we aggregated all REACT data to the national level, estimated the *δ* curve at this ultra-coarse national level and then took this *δ* forward to estimate local prevalence. We found that estimates retained a high level of accuracy (Extended Data Fig. 4g–i). Second, to examine the effects of a more limited randomized surveillance regime, we left out REACT round 8, re-estimated *δ* curves at the PHE-region level and used these to infer local prevalence. In this case, we lost precision in our prevalence estimates for omitted round 8, as we would expect, but the estimates remained highly accurate, with average bias of 0.05% (SE = 0.03; see also Extended Data Fig. 4j–l and compare vertical credible interval widths between Extended Data Fig. 4e and Extended Data Fig. 4k).

### Predictive ability of *R*_*t*_ estimates

**Extended Data Fig. 5:**
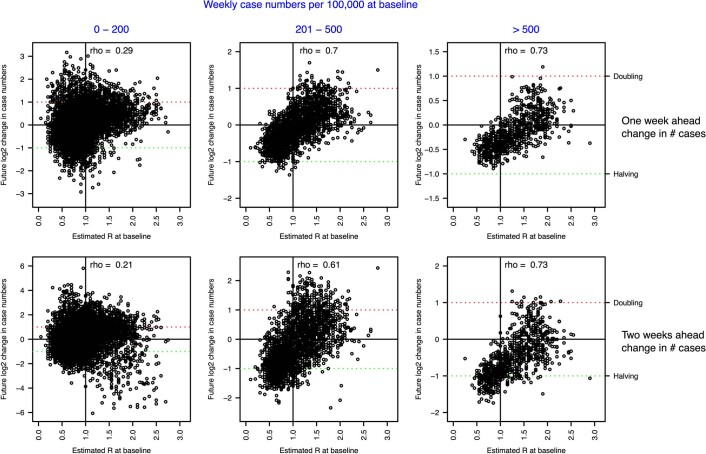
Predicting future change in case numbers from current estimated ${{{{\mathcal{R}}}}}_{t}$. Each point corresponds to an (LTLA, week) pair, predicting future case numbers in the LTLA using ${{{{\mathcal{R}}}}}_{t}$ for that week. Future case numbers are represented by forward-in-time ${{{\mathrm{log}}}\,}_{2}$ fold change ${{{\mathrm{log}}}\,}_{2}({n}_{t+k}/{n}_{t})$. Case data underlying the plot are from the period 2020-10-18 - 2021-06-20. Note the number of points in each column differs based on how many LTLA-week pairs have baseline case numbers in the intervals in blue shown at the top of the plot.

*R*_*t*_ measures whether the number of infectious individuals is increasing, *R*_*t*_ > 1, or decreasing, *R*_*t*_ < 1, in the population at time point *t*. Extended Data Fig. 5 compares LTLA *R*_*t*_ estimates with the future change in local case numbers. For validation purposes, here we are performed one-step-ahead at a time prediction and compared predictions with out-of-training-sample observed statistics (fold-change in raw case numbers from baseline). The results were stratified according to baseline case numbers, and we examined predictions 1 week and 2 weeks ahead. Each point corresponds to an (LTLA, week) pair, and the results are for the period 18 October 2020 to 20 June 2021. Across each of the six scenarios presented, there is strong evidence of an association between *R*_*t*_ and future change in case numbers (*P* < 2 × 10^−16^). The strength of association between *R*_*t*_ and 1-week-ahead case numbers has Spearman’s *ρ* = 0.73 for the high baseline case group (>500 cases per 100,000), which decreased to *ρ* = 0.29 in the low baseline group (≤200 cases per 100,000). The association remained strong when predicting caseloads 2 weeks ahead, with, for example, *ρ* = 0.73 (Spearman’s) for the high baseline case group.

### Comparison of effective reproduction number estimates from the debiasing approach with estimates from other studies

**Extended Data Fig. 6:**
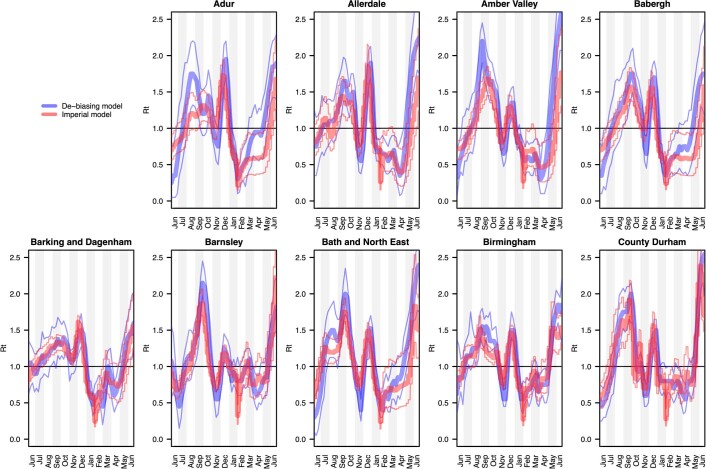
Comparison of ${{{{\mathcal{R}}}}}_{t}$ estimates between de-biasing model and Imperial model. For each of the nine PHE regions, we present the constituent LTLA whose name is ranked top alphabetically.

We extracted estimates of *R*_*t*_ based on our debiasing model likelihood implemented within a standard SIR model, illustrated in Extended Data Fig. 1. We compared the results to the local *R*_*t*_ estimates outputted by at the Imperial College COVID-19 website^32^. A cross-method comparison of longitudinal traces of *R*_*t*_ for a subset of LTLAs is shown in Extended Data Fig. 6. Encouragingly for both approaches, the estimates generally displayed good concordance, with credible intervals overlapping appropriately, despite being based on different data and models (Supplementary Table 1).

## Discussion

The current standard practice internationally is to summarize SARS-CoV-2 infection rates by counting the number of individuals testing positive in a local area over a period of time, typically 1 week. The resulting statistic—cases per 100,000—is used to characterize and monitor the spatiotemporal state of an epidemic alongside other epidemiological measures such as *R*_*t*_. Problematically, however, interpreting cases per 100,000 is not straightforward, as the data are subject to a number of unknown biasing influences such as (1) variation in testing capacity, (2) ascertainment bias on who is (self)-selected to be tested and (3) imperfect sensitivity and specificity of antigen tests. These factors, among others, make it difficult to quantify the true underlying local incidence or prevalence of SARS-CoV-2 infection, which places a burden on policymakers implicitly to adjust for such biases themselves. To address this problem, we developed an integrative causal model that can be used to debias raw case numbers and accurately estimate the number of individuals with infection in a local area.

The flexible statistical framework allows simultaneous and coherent incorporation of a number of important features. First, it corrects for ascertainment bias that result from preferential testing based on symptom status or on other confounders. This accounts for any variation in testing capacity by modelling the total number of tests conducted locally. Second, it can incorporate the use of different SARS-CoV-2 testing assays, such as LFD and PCR, including adjustment for particular sensitivity and specificity. Third, it infers the number of infectious individuals, while PCR tests may also pick up positive individuals at non-infectious stages. Finally, the model outputs week-specific debiased prevalence with uncertainty (via a marginal likelihood), which allows modular interoperability with other models. We illustrated this with a SIR epidemic model implementation that estimated local transmission rates while accounting for vaccine- and disease-induced immunity in the population. Our modelling work illustrates the benefits of having both a rolling randomized surveillance survey and targeted testing (for example, of frontline healthcare staff and symptomatic individuals). While targeted testing is routinely collected internationally, the United Kingdom has led the way in introducing regular national surveillance randomized surveys such as REACT^7,8^ and ONS CIS^6^. Ongoing international pandemic preparedness can benefit from sampling designs that combine random sampling with targeted testing so that they can most powerfully complement and strengthen one another. Our model depends on the availability of randomized surveillance data. Future studies from other countries and collaborations with local experts will show and may further validate the breadth of utility of our debiasing framework and how it contributes towards global public health responses.

Since randomized surveillance data are currently rare internationally, there would be utility in extending the causal framework to address situations where targeted testing is accompanied by semi-randomized data with a well-known selection process (such as routine tests for healthcare workers, in care homes or regular testing at schools). Extending the current framework would begin with careful empirical exploration of the relationship between test positivity rate in such semi-randomized settings and comparable local prevalence (for example, in relevant age strata). The wealth of data available in the United Kingdom provides a good starting point for such exploratory work, which can be used to develop more complex causal models transferable to new semi-randomized contexts.

## Methods

### Ethics approval

The Alan Turing Institute Ethics Advisory Group provided guidelines for this study’s procedures and advised that Health Research Authority approval is not required for this research.

### Observational models for surveillance data

The primary target of inference is prevalence, *I* out of *M*, being the unknown number of infectious individuals at a particular time point in the local population of known size *M*. Our method estimates two types of prevalence: (1) the number of individuals that would test PCR positive ($\tilde{I}$) and (2) the number of individuals that are infectious (*I*). See below (“Focusing prevalence on the infectious subpopulation”), where we clarify the distinction between the PCR-positive and infectious subpopulations, and how we target the latter.

#### Temporal resolution of test count data

We applied the debiasing framework to test-count data aggregated into non-overlapping weeks. This has two clear advantages. First, by aggregating to weekly level data, we obviate the need to account for weekday effects that can be driven, for example, by logistical constraints or by individuals self-selecting to submit samples more readily on some weekdays than on others. Second, fitting a weekly model is computationally less intensive than fitting a model to daily test counts. The potential disadvantage of binning data by week is that high-frequency effects cannot be detected. Although it is possible in principle to adapt the framework to analyse daily testing data, we note that daily variation is likely to be confounded by weekday testing effects and so may be difficult to detect and interpret. Furthermore, while we use non-overlapping weekly data for model fitting, it is possible to output rolling weekly estimates, particularly to obtain as up-to-date prevalence estimates as are permitted by the data. However, we note that complete testing data are typically subject to a reporting lag of 4–5 days^33^.

#### Randomized surveillance data, *u* of *U*

Suppose that out of a total *U* randomized surveillance (for example, REACT and ONS CIS) tests, we observe *u* positive tests. The randomized testing (for example, REACT and ONS CIS) likelihood is

2 $${\mathbb{P}}(u\,{{\mbox{of}}}\,U\,|\, \tilde{I})={{{\rm{Hypergeometric}}}}(u\,|\, M,\tilde{I},U)\,,$$

and this allows direct, accurate statistical inference on $\tilde{I}$, the proportion of the population that would return a positive PCR test.

#### Focusing prevalence on the infectious subpopulation

PCR tests are sensitive and can detect the presence of SARS-CoV-2 both days before and weeks after an individual is infectious. It is usually desirable for prevalence to represent the proportion of a population that is infectious. We can obtain a likelihood for the number of infectious individuals *I* as follows:

3 $${\mathbb{P}}(n\,{{\mbox{of}}}\,N\,|\, I)={\int}\ {\mathbb{P}}(n\,{{\mbox{of}}}\,N\,|\, \tilde{I}){\mathbb{P}}(\tilde{I}\,|\, I){\mathrm{d}}\tilde{I},$$

where *I* and $\tilde{I}$ are the number of infectious and PCR-positive individuals, respectively.

The conditional distribution ${\mathbb{P}}(\tilde{I}\,|\, I)$ can be specified on the basis of external knowledge of the average length of time spent PCR-positive versus infectious. Our approach to estimating this quantity imports information on the timing of COVID-19 transmission^34^ and the interval of PCR positivity in individuals with SARS-CoV-2 infection^35^. More precisely, we specified the infectious time interval for an average individual with infection in the population to span the interval 1–11 days after infection (the empirical range of generation time from fig. 1A of ref. ^34^). We then calculated the posterior probability of a positive PCR occurring 1–11 days after infection (fig. 1A of ref. ^35^). We incorporated the effects of changing incidence in the calculations; this is important because, for example, if incidence is rising steeply, the majority of people who would test PCR positive in the population are those that are relatively recently infected. Full details can be found in Supplementary Information “PCR positive to infectious mapping—method details”.

#### Targeted surveillance data, *n* of *N*

In contrast to the randomized surveillance likelihood in equation (2), the targeted likelihood can be expressed in terms of the observation of *n* of *N* positive targeted (for example Pillar 1+2) tests as follows:

4 $$\begin{array}{ll}{\mathbb{P}}(n\,{{\mbox{of}}}\,N\,|\, I,\delta ,\nu )&={{{\rm{Binomial}}}}\left(n\,|\, I,\,{\mathbb{P}}(\,{{\mbox{tested}}}\,|\, {{\mbox{infected}}}\,)\right)\,\\ &\times \,{{{\rm{Binomial}}}}(N-n\,|\, M-I,\,{\mathbb{P}}(\,{{\mbox{tested}}}\,|\, {{\mbox{not}}}\,{{\mbox{infected}}}\,)),\end{array}$$

where ${\mathbb{P}}(\,{{\mbox{tested}}}\,|\, {{\mbox{infected}}}\,)$ and ${\mathbb{P}}({{\mbox{tested}}}\,|\, {{\mbox{not}}}\,{{\mbox{infected}}})$ are the probabilities of an individual with infection (respectively, individual without infection) being tested.

#### Bias parameters, *δ* and *ν*

We introduce the following parameters:

5 $$\delta :={{\mathrm{log}}}\,\left(\frac{{{{\rm{odds}}}}(\,{{\mbox{tested}}}\,|\, {{\mbox{infected}}}\,)}{{{{\rm{odds}}}}(\,{{\mbox{tested}}}\,|\, {{\mbox{not}}}\,{{\mbox{infected}}}\,)}\right)$$

6 $$\nu :={{\mathrm{log}}}\,{{{\rm{odds}}}}(\,{{\mbox{tested}}}\,|\, {{\mbox{not}}}\,{{\mbox{infected}}}\,)\,,$$

leading to the targeted swab testing likelihood being represented as

7 $$\begin{array}{ll}{\mathbb{P}}(n\,{{\mbox{of}}}\,N\,|\, I,\delta ,\nu )=&{{{\rm{Binomial}}}}\left(n\,|\, I,\,{{{{\rm{logit}}}}}^{-1}(\delta +\nu )\right)\,\\ &\times \,{{{\rm{Binomial}}}}(N-n\,|\, M-I,\,{{{{\rm{logit}}}}}^{-1}\nu )\,.\end{array}$$

The unknown parameter that requires special care to infer is *δ*, that is, the log odds-ratio of being tested in the infected subpopulation versus in the non-infected subpopulation. The other parameter, *ν*, is directly estimable from the targeted data: $\hat{\nu }:=\,{{\mbox{logit}}}\,[(N-n)/M]$ is a precise estimator with little bias when prevalence is low.

#### Test sensitivity and specificity

The likelihood in equation (7) assumes a perfect antigen test. If the test procedure has false-positive rate *α*, and false-negative rate *β*, the targeted likelihood is instead

8 $${\mathbb{P}}(n\,{{{\rm{of}}}}\,N\,|\, I,\delta ,\nu )=\mathop{\sum }\limits_{z=0}^{\min \{I,N\}}{\mathbb{P}}(z\,{{{\rm{of}}}}\,N\,|\, I,\delta ,\nu ){\mathbb{P}}(n\,|\, z\,{{{\rm{of}}}}\,N)\,\,,$$

where *z* denotes the unknown number of individuals who truly have an infection that were tested. The first term in the sum in equation (8) is obtained by substituting *z* in equation (7), while the second term is

9 $${\mathbb{P}}(n\,|\, z\,{{{\rm{of}}}}\,N)=\mathop{\sum }\limits_{{n}_{\beta }=\max \{0,z-n\}}^{\min \{z,N-n\}}{{{\rm{Binomial}}}}({n}_{\beta }\,|\, z,\,\beta )\,{{{\rm{Binomial}}}}({n}_{\beta }+n-z\,|\, N-z,\,\alpha )\,,$$

with *n*_*β*_ denoting the number of false-negative test results. An analogous adjustment can be made to the randomized surveillance likelihood in equation (2).

### Cross-sectional inference on local prevalence

We leveraged spatially coarse-scale randomized surveillance data to specify an EB prior on bias parameters *p*(*δ*) at coarse-scale (PHE region), and thereby accurately infer prevalence from targeted data at fine scale (LTLA *j* within PHE region *J*_*j*_). We explicitly use the superscripts LTLA (*j*) in PHE region (*J*_*j*_) in step 4 below, where notation from both coarse and fine scale appear together. All quantities in steps 1–3 are implicitly superscripted (*J*_*j*_), but these are suppressed for notational clarity. For computational efficiency, we handle prevalence in a reduced-dimension space of bins as described in Supplementary Information section “Interval-based prevalence inference—set-up and assumptions”. The method in detail is as follows:

1. Infer prevalence from unbiased testing data. At a coarse geographic level (PHE region *J*_*j*_), estimate prevalence from randomized surveillance data *u*_*t*_ of *U*_*t*_. Represent the posterior at time *t* in mass function
  10 $${\hat{p}}_{t}({I}_{t}):={\mathbb{P}}({I}_{t}\,|\, {u}_{t}\,{{\mbox{of}}}\,{U}_{t})$$
  where ${\hat{p}}_{t}:\{0,\ldots ,{{{\rm{M}}}}\}\to [0,1]$ need only be available at a subset $t\in {{{\mathcal{T}}}}\subseteq \{1,\ldots ,T\}$ of time points.
2. Learn *δ*_*t*_ from accurate prevalence. At a coarse geographic level, for each $t\in {{{\mathcal{T}}}}$, we estimate bias parameter *δ*_*t*_ by coupling biased data *n*_*t*_ of *N*_*t*_ with accurate prevalence information ${\hat{p}}_{t}$. With *ν*_*t*_ fixed at $\hat{{\nu }_{t}}:=\,{{\mbox{logit}}}\,[({N}_{t}-{n}_{t})/M]$
  11 $$p({\delta }_{t}\,|\, {n}_{t}\,{{\mbox{of}}}\,{N}_{t},{\hat{p}}_{t},\hat{{\nu }_{t}})=\mathop{\sum}\limits_{{I}_{t}}p({\delta }_{t}\,|\, {n}_{t}\,{{\mbox{of}}}\,{N}_{t},{I}_{t},\hat{{\nu }_{t}}){\hat{p}}_{t}({I}_{t})$$
  12 $$\approx \,{{\mbox{N}}}\,({\delta }_{t}\,|\, {\hat{\mu }}_{t},\,{\hat{\sigma }}_{t}^{2})$$
  where a moment-matched Gaussian approximation is performed in equation (12) (we assessed the reasonableness of this approximation using diagnostic plots (Supplementary Fig. 2)). The posterior density in the sum in equation (11), $p({\delta }_{t}\,|\, {n}_{t}\,{{\mbox{of}}}\,{N}_{t},{I}_{t},\hat{{\nu }_{t}})$ is conjugate under a Beta(*a*,*b*) prior on ${{{\mbox{logit}}}}^{-1}({\nu }_{t}+{\delta }_{t})\equiv {\mathbb{P}}(\,{{\mbox{tested}}}\,|\, {{\mbox{infected}}}\,)$, and so can be evaluated as follows (where BetaCDF is the cumulative distribution function of the beta distribution):
  13 $${\mathbb{P}}({\delta }_{t}\le \,{{\mbox{logit}}}\,(x)-\hat{{\nu }_{t}}\,|\, {n}_{t}\,{{\mbox{of}}}\,{N}_{t},{I}_{t},\hat{{\nu }_{t}})=\,{{\mbox{BetaCDF}}}\,(x\,|\, {n}_{t}+a,{I}_{t}-{n}_{t}+b)\,.$$
3. Specify smooth EB prior on *δ*_1:*T*_. A smooth prior on *δ*_1:*T*_ is specified as follows:
  14 $$p({{\boldsymbol{\delta}}})\propto {{{\rm{N}}}}({{{\boldsymbol{\delta} }}}\,|\, {{{\boldsymbol{0}}}},{{{{{\Sigma }}}}}_{\delta })\,\mathop{\prod}\limits_{t\in {{{\mathcal{T}}}}}{{{\rm{N}}}}({\delta }_{t}\,|\, {\hat{\mu }}_{t},{\hat{\sigma }}_{t}^{2})\mathop{\prod}\limits_{t\notin {{{\mathcal{T}}}}}{{{\rm{N}}}}({\delta }_{t}| 0,{\sigma }_{\small{\,{{\mbox{flat}}}\,}}^{2})$$
  where N(***δ*** ∣ **0**, **Σ**_*δ*_) imparts a user-specified degree of longitudinal smoothness, thereby sharing information on *δ* across time points. Ignorance of *δ*_*t*_, in the absence of random surveillance data, is encapsulated in a Gaussian with large variance $\sigma_{\small{\,{{\mbox{flat}}}\,}}^{2}$. A standard choice for N(***δ*** ∣ **0**, **Σ**_*δ*_) corresponds to a stationary autoregressive, AR(1), process of the form
  15 $${\delta }_{t}=c+\psi {\delta }_{t-1}+{\varepsilon }_{t}$$
  with a diffuse Gaussian prior $c \sim {{{\rm{N}}}}(0,{\sigma }_{\,{\small{{\mbox{flat}}}}\,}^{2})$ and with smoothing tuned by 0 < *ψ* < 1 and white noise variance ${\sigma }_{\varepsilon }^{2}$. The normalized form of the prior in equation (14) is
  16 $$p({{\boldsymbol{\delta} }})={{{\rm{N}}}}\left({{\boldsymbol{\delta} }}\,| \,{({{{{\boldsymbol{\Sigma }}}}}_{\delta }^{-1}+{{D}}^{-1})}^{-1}{{D}}^{-1}{{{\hat{\boldsymbol{\mu} }}}},\,{({{{{\boldsymbol{\Sigma }}}}}_{\delta }^{-1}+{{D}}^{-1})}^{-1}\right)$$
  with ($\hat{\mu}$, diagonal matrix *D*_*T*×*T*_) having elements $({\hat{\mu }}_{t},{\hat{\sigma }}_{t}^{2})$ for $t\in {{{\mathcal{T}}}}$ and $(0,{\sigma }_{\,{\small{\mbox{flat}}}\,}^{2})$ for $t\notin {{{\mathcal{T}}}}$.
4. Infer cross-sectional local prevalence from biased testing data. At a fine-scale geographic level (LTLA *j* in PHE region *J*_*j*_), having observed ${n}_{t}^{(j)}\,{{\mbox{of}}}\,{N}_{t}^{(j)}$ positive test results (a subset of the ${n}_{t}^{({J}_{j})}\,{{\mbox{of}}}\,{N}_{t}^{({J}_{j})}$ observed at the coarse-scale level above), we calculated the posterior for ${I}_{t}^{(j)}$ separately at each time point *t* as follows:
  17 $$p({I}_{t}^{(j)}| {n}_{t}^{(j)}\,{{\mathrm{of}}}\ {N}_{t}^{(j)})\propto p({I}_{t}^{(j)})p({n}_{t}^{(j)}{{\mathrm{of}}}\ {N}_{t}^{(j)}| {I}_{t}^{(j)},{\hat{\nu }}_{t}^{(j)})$$
  18 $$=p({I}_{t}^{(j)}){\int}_{{\delta }_{t}^{({J}_{j})}}p({n}_{t}^{(j)}\,{{\mathrm{of}}}\ {N}_{t}^{(j)}| {I}_{t}^{(j)},{\hat{\nu }}_{t}^{(j)},{\delta }_{t}^{({J}_{j})})p({\delta }_{t}^{({J}_{j})})d{\delta }_{t}^{({J}_{j})}$$
  where ${\hat{\nu }}_{t}^{(j)}:=\,{{\mbox{logit}}}\,[({N}_{t}^{(j)}-{n}_{t}^{(j)})/{M}_{t}^{(j)}]$, the likelihood in the integral in equation (18) is available in equation (7), and the prior $p({\delta }_{t}^{({J}_{j})})$ is time point *t*’s marginal Gaussian from equation (16).

#### Debiasing LFD tests with PCR surveillance (or vice versa)

The methods can be adapted in a straightforward manner to the situation in which the randomized surveillance study uses a different assay to the targeted testing. For a concrete example, we could use REACT PCR prevalence posterior ${\hat{p}}_{t}({\tilde{I}}_{t})$ from equation (10) to debias Pillar 1+2 LFD test data *n*_*t*_ of *N*_*t*_. Equation (11) can be adjusted to estimate the ascertainment bias *δ* pertaining to LFD data as follows:

19 $$p({\delta }_{t}\,|\, {n}_{t}\,{{\mbox{of}}}\,{N}_{t},{\hat{p}}_{t},\hat{{\nu }_{t}})=\mathop{\sum}\limits_{{\bar{I}}_{t}}\{p({\delta }_{t}\,|\, {n}_{t}\,{{\mbox{of}}}\,{N}_{t},{\bar{I}}_{t},\hat{{\nu }_{t}})\mathop{\sum}\limits_{{\tilde{I}}_{t}}{\mathbb{P}}({\bar{I}}_{t}\,|\, {\tilde{I}}_{t}){\hat{p}}_{t}({\tilde{I}}_{t})\}\,,$$

where ${\bar{I}}_{t}$ and ${\tilde{I}}_{t}$ are the unobserved LFD- and PCR-positive prevalence, respectively, and the conditional distribution ${\mathbb{P}}({\bar{I}}_{t}\,|\, {\tilde{I}}_{t})$ can be estimated on the basis of external knowledge of the average length of time spent PCR-positive versus LFD-positive, analogously to as described in above in “Focusing prevalence on the infectious subpopulation”. The remaining computations, from equation (12) onwards, are unchanged, with the outputted fine-scale marginal likelihood $p({n}_{t}^{(j)}\,{{\mbox{of}}}\,{N}_{t}^{(j)}\,|\, {I}_{t}^{(j)},{\hat{\nu }}_{t}^{(j)})$ in equation (17) to be interpreted as targeting the local LFD-positive prevalence ${\bar{I}}_{t}^{(j)}$.

### Full Bayesian inference under a stochastic SIR epidemic model

The cross-sectional analysis described above in “Cross-sectional inference on local prevalence” generates the *δ*-marginalized likelihood, $p({n}_{t}^{(j)}\,{{\mbox{of}}}\,{N}_{t}^{(j)}\,|\, {I}_{t}^{(j)},{\hat{\nu }}_{t})$ in equation (17), at each time point for which targeted data are available. These likelihoods can be used as input for longitudinal models to obtain better prevalence estimates and to infer epidemiological parameters such as *R*_*t*_.

We illustrate this via a Bayesian implementation of a stochastic epidemic model whereby individuals become immune through population vaccination and/or exposure to COVID-19 (Supplementary Fig. 1). We incorporate known population vaccination counts into a standard discrete time Markov chain SIR model (ref. ^36^, chapter 3). Details of the transition probability calculations are given in the Supplementary Information sections “SIR model details” and “SIR model—discussion, assumptions and caveats”.

#### Priors on *R*, *I* and *R*^+^

We place priors on *I*, *R*^+^ measured as a proportion of the population; this proportion then gets mapped to prevalence intervals on subpopulation counts as described in “Interval-based prevalence inference—set-up and assumptions” in the Supplementary Information. Specifically, we use truncated, discretized Gaussian distributions on the proportion of the population who are immune and infectious. For example, on the number of infectious individuals *I*_*t*_ at each time point *t*, we specify the prior (suitably normalized over its support)

20 $${\mathbb{P}}({I}_{t}=j)\propto \int\nolimits_{(j-1)/M}^{j/M}{{{\rm{N}}}}\left(x| {\mu }_{I},{\sigma }_{I}^{2}\right){\mathrm{d}}x\,\,\,{{{\rm{for}}}}\,\,\,j/M\in [{p}_{\min },\ldots ,{p}_{\max }]\,,$$

with an example weakly informative hyperparameter setting being ${\mu }_{I}=0.5 \% ,\,{\sigma }_{I}=1 \% ,\,{p}_{\min }=0 \% ,\,{p}_{\max }=4 \%$. To ensure meaningful inference on ${R}_{1:T}^{+}$, we placed an informative prior that reflects the state of knowledge of the immune population size. We did this using an informative truncated Gaussian prior on ${R}_{1}^{+}$ and noninformative priors on ${R}_{2:T}^{+}$. We placed a noninformative uniform prior on each *R*_*t*_, for example a Uniform(0.5, 2.5).

#### Markov chain Monte Carlo sampling implementation

We performed inference under the model represented in the DAG in Supplementary Fig. 1. The likelihood is marginalized with respect to *δ*, and we used Markov chain Monte Carlo to draw samples from the posterior

$$p({{I}},{{{R}}}^{+},{{{\mathcal{R}}}}\,|\, {{n}},{{N}})\,.$$

We sampled $\mathcal{R}$ and (*I*, *R*^+^) using separate Gibbs updates. For sampling (*I*, *R*^+^), we represented the joint full conditional as

21 $$p({I},{R}^{+}| {\mathcal{R}},{n},{N})=p({I}\,| \,{\mathcal{R}},{n},{N})p({R}^{+}| {I})\,,$$

sampling *I*^new^ from $p({I}\,|\, {\mathcal{R}},{n},{N})$, and then ${R}^{{+}^{{{\mbox{new}}}}}$ from *p*(*R*^+^ ∣ *I*^new^).

#### Sampling from *p*(*I* | *𝑅*, *n*, *N*)

The sampling distribution on prevalence can be expressed as

22 $$\begin{array}{ll}p({I}\,|\, {\mathcal{R}},{n},{N})&\propto p({n},{N}\,|\, {I},{\mathcal{R}})p({I}\,|\, {\mathcal{R}})\\ &=p({n}_{1},{N}_{1}\,|\, {I}_{1})p({I}_{1})\mathop{\prod }\limits_{t=2}^{T}p({n}_{t}\,{{\mbox{of}}}\,{N}_{t}\,|\, {I}_{t})p({I}_{t}\,|\, {I}_{t-1},{{{{\mathcal{R}}}}}_{t-1}),\end{array}$$

which is a hidden Markov model with emission probabilities taken from the *δ*-marginalized likelihood in equation (18), and transition probabilities taken from equation (37) (Supplementary Information).

#### Sampling from *p*(*R*^+^∣*I*)

We expressed the full conditional for ${{\Delta }}\ {R}_{1:T}^{+}$ as

$${\mathbb{P}}({R}_{1:T}^{+}\,|\, {I}_{1:T})\propto {\mathbb{P}}({R}_{1}^{+}\,|\, {V}_{1})\mathop{\prod }\limits_{t=2}^{T}{\mathbb{P}}({R}_{t}^{+}\,|\, {R}_{t-1}^{+},{I}_{t-1},{{\Delta }}\ {V}_{t})$$

and sampled the ${{\Delta }}\ {R}_{1:T}^{+}$ sequentially, with ${\mathbb{P}}({R}_{t}^{+}\,|\, {R}_{t-1}^{+},{I}_{t-1},{{\Delta }}\ {V}_{t})$ available in equation (39) (Supplementary Information).

#### Sampling from *p*(*𝑅* | *I*)

The prior joint distribution of ${{{{\mathcal{R}}}}}_{1:T}$ was modelled using a random walk as follows:

23 $${\mathcal{R}}_{t} \sim {{{\rm{N}}}}({{{{\mathcal{R}}}}}_{t-1},\,{\sigma }_{{{{\mathcal{R}}}}}^{2})\,,$$

where ${\sigma }_{{{{\mathcal{R}}}}}^{2}$ is a user-specified smoothness parameter.

The update involves sampling from

24 $$p({\mathcal{R}}\,|\, {I})=p({{{{\mathcal{R}}}}}_{1})\mathop{\prod }\limits_{t=2}^{T-1}p({{{{\mathcal{R}}}}}_{t}\,|\, {{{{\mathcal{R}}}}}_{t-1})\mathop{\prod }\limits_{t=2}^{T}p({I}_{t}\,|\, {I}_{t-1},{{{{\mathcal{R}}}}}_{t-1})\,.$$

We discretized the space of *R*_*t*_ into an evenly spaced grid and sample from the hidden Markov model defined in equation (24)^37^. The transition probabilities are given by equation (23) (suitably normalized over the discrete *R*_*t*_ space) and the emission probabilities given by equation (37) (Supplementary Information).

### Reporting Summary

Further information on research design is available in the Nature Research Reporting Summary linked to this article.

## Supplementary information

Supplementary Information Supplementary Figs. 1–7, Supplementary Table 1, Discussion of methodological assumptions and caveats, Supplementary Results.

Reporting Summary

## Data Availability

The data underlying the Alpha VoC 202012/01 analysis were accessed via the UK Health Security Agency Data Science Hub (DaSH) data platform; they are not publicly available and can only be accessed using approved UK government email domains such as @test-and-trace.nhs.uk. For the remainder of the results presented here, the data are publicly available. Randomized surveillance data comes from the REACT study^7,8^ (https://github.com/mrc-ide/reactidd/tree/master/inst/extdata). From REACT, we create weekly test counts at the spatially coarse-scale level (PHE region) and, for validation purposes but not model fitting, use round-aggregated counts at the fine-scale level (LTLA), for rounds 7–11. The combined weekly Pillar 1+2 data are publicly available for download (https://www.gov.uk/government/publications/nhs-test-and-trace-england-statistics-14-january-to-20-january-2021; note that LFD results are not included in these weekly summaries). We downloaded *R*_*t*_ estimates outputted by the Imperial College team’s Epidemia model^38,39^ from https://imperialcollegelondon.github.io/covid19local/downloads/UK_hotspot_Rt_estimates.csv on 13 October 2021, and we provide a copy of that downloaded file in our Zenodo repository at 10.5281/zenodo.5784718.

## Code availability

The R scripts^40^ used to generate the results in this manuscript are available in the following Git repository: https://github.com/alan-turing-institute/jbc-turing-rss-testdebiasing.
