# Supplementary material for: Improving local prevalence estimates of SARS-CoV-2 infections using a causal debiasing framework
Source: Nat Microbiol. 2021 Dec 31;7(1):97–107. doi: 10.1038/s41564-021-01029-0 (PMC8727294; doi:10.1038/s41564-021-01029-0)
Supplement: Supplementary file 1 — Supplementary Figs. 1–7, Supplementary Table 1, Discussion of methodological assumptions and caveats, Supplementary Results. [file 41564_2021_1029_MOESM1_ESM.pdf]

nature portfolio

https://doi.org/10.1038/s41564-021-01029-0

Supplementary information

Improving local prevalence estimates of SARS-CoV-2 infections using a causal debiasing framework

In the format provided by the authors and unedited

Supplementary Table 1: Details of related work

Authors	Data inputs	Method overview	Outputs
Birrell *et al.* Medical Research Council (MRC) Biostatistics Unit (BSU), PHE [18]	Daily death and serological data at PHE region level. ONS CIS data.	Age-stratified ordinary differential equation (ODE)-based transmission models in each of the seven NHS regions of England, with regional epidemics sharing common parameters, and estimation carried out in a Bayesian framework	Daily, PHE regional $\mathcal{R}_t$
Irons and Raftery [19]	Deaths and case count data in the United States (including some randomised surveillance data)	Bayesian framework built on an empirical functional relationship between cumulative under-reporting of cases and cumulative number of tests conducted, allowing inference on cumulative incidence and SIR epidemic model fitting	Infection-to-fatality ratio, cumulative incidence and $\mathcal{R}_t$ at US state-wide level
Teh *et al.*, Data Evaluation and Learning for Viral Epidemics (DELVE) [20]	Pillar 1+2 daily case data across England, Wales and Scotland. Commuter-flow data.	Hierarchical Bayesian method for estimating local $\mathcal{R}_t$, modelling both temporal and spatial dependence in transmission rates, and based on renewal equations [21, 22]	Local LTLA-level estimates and predictions of $\mathcal{R}_t$ and positive case numbers
Jewell *et al.* [23]	Pillar 1+2 case data across England. Human mobility data.	Bayesan implementation of an Susceptible-Exposed-Infectious-Removed (SEIR) model allowing for transmission within and betweem local authority districts (LADs)	Daily case prevalence (proportion of infected population), incidence, and $\mathcal{R}_t$
Mishra *et al.*, Epidemia [38, 32, 39, 22]	Daily UK case data; weekly deaths data; daily randomized surveillance outputs from the ONS CIS and REACT studies	Modified version of the Bayesian semi-mechanistic model of [22] calibrated through the infection fatality ratio and infection ascertainment rate estimated from national-level randomised surveillance data	Estimates and predictions of $\mathcal{R}_t$, positive case numbers, and change in new infections
Colman *et al.* [24]	Pillar 1+2 case counts across England's PHE regions. ONS CIS data.	Targets the proportion of infections that result in a positive diagnosis, comprising time-dependent test sensitivity and the proportion of infected individuals who seek testing, with estimation calibrated against surveillance data at PHE region level	Daily incidence of infections over time at a PHE region level
Abbott *et al.* centre for mathematical modelling of infectious diseases (CMMID) COVID modelling group. [25]	Case counts and death notifications	Bayesian renewal equation approach based on [21] allowing for uncertainty in delay between onset and case report or death	Daily estimates of $\mathcal{R}_t$ at a global, national and subnational (PHE regional) level
Nicholson *et al.* The Alan Turing Institute and Royal Statistical Society Statistical Modelling and Machine Learning Laboratory (Current work)	Pillar 1+2 weekly positive and total counts at glsltla level; REACT weekly positive and total counts at PHE regional level	Ascertainment bias estimated from REACT data at PHE region level and used to infer prevalence from positive and total test counts at the local LTLA level. SIR model used to infer $\mathcal{R}_t$	Weekly estimates of point prevalence and $\mathcal{R}_t$ at LTLA level

Stochastic epidemic model

The DAG for the stochastic epidemic model whereby individuals become immune through population vaccination and/or exposure to COVID-19 is shown in Supplementary Fig. 1).

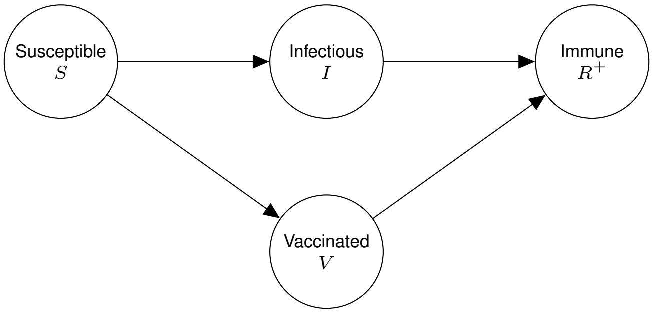

Supplementary Figure 1: SIR/V epidemic model compartmental diagram.

Model parameters

A full list of model parameters, along with either their prior distribution or the value at which they were fixed, can be found in Supplementary Table 2.

Supplementary Table 2: Model parameters with specified prior distributions or fixed values

**Parameter**	**Prior / Fixed value**
Ascertainment bias, $\delta_{1:T}$	Empirical Bayes prior (see Eq. (16)):
	- AR(1) coefficient, $\psi = 0.99$
	- Standard deviation, $\sigma_\epsilon = 1$
	- Intercept, $c \sim \mathcal{N}(0, \sigma^2_{flat})$ with $\sigma_{flat} = 10$
PCR false-positive rate, α	Fixed, $\alpha = 3 \times 10^{-4}$ (see Eq. (58) and Supplementary Fig. 5)
PCR false-negative rate, β,	Fixed, $\beta = 0.05$, taken from [40]
Expected time to recovery, $1/\gamma$	$T_{\text{recovery}} \sim$ Exponential(γ), with $\gamma = 1$ week
Effective reproduction number, $\mathcal{R}_t$	Random walk: $\mathcal{R}_t \sim \mathcal{N}(\mathcal{R}_{t-1}, \sigma^2_{\mathcal{R}})$, with $\sigma_{\mathcal{R}} = 0.2$
Proportion immune at $t = 0$, R^+_0/M	Truncated Gaussian (see Eq. (20), and reference [41])
	- Mean, $\mu_R = 0.06$
	- Standard deviation, $\sigma_R = 0.01$
	- Minimum proportion, $p_{\min} = 0$
	- Maximum proportion, $p_{\max} = 0.1$
Proportion infectious at each t, I_t/M	Truncated Gaussian (see Eq. (20)):
	- Mean, $\mu_I = 0.005$
	- Standard deviation, $\sigma_I = 0.01$
	- Minimum proportion, $p_{\min} = 0$
	- Maximum proportion, $p_{\max} = 0.04$

Discussion of methodological assumptions and caveats

Interval-based prevalence inference – set-up and assumptions

The full prevalence state space comprises all potential numbers of infectious individuals in the population, i.e. $I \in \{0, \ldots, M\}$. For computational tractability we define $B \ll M$ bins:[3]

$$\mathcal{B}_b \quad := \quad \{I : e_{b-1} \leq I < e_b\} \quad b = 1, \ldots, B \tag{25}$$

having midpoints:

$$\check{I}_b \quad := \quad \left\lfloor \frac{e_{b-1} + e_b - 1}{2} \right\rfloor \ , \ b = 1, \ldots, B \ , \tag{26}$$

and make three assumptions to allow computationally efficient inference on the B-dimensional space of bins, denoting these assumptions Interval-1:3 as follows:

Interval-1 The testing data likelihood, conditional on prevalence bin, is evaluated at the bin midpoint:

$$\mathbb{P}(n \text{ of } N, u \text{ of } U \mid I \in \mathcal{B}_b) \quad := \quad \mathbb{P}(n \text{ of } N, u \text{ of } U \mid I = \check{I}_b) \ . \tag{27}$$

Interval-2 Prevalence I is uniformly distributed within each bin:

$$\mathbb{P}(I = k \mid I \in \mathcal{B}_b) \quad := \quad \begin{cases} \frac{1}{e_b - e_{b-1}} & k \in \mathcal{B}_b \\ 0 & \text{otherwise.} \end{cases} \tag{28}$$

Interval-3 The distribution of new infections, conditional on prevalence bin, is evaluated at the bin midpoint (with the same assumption applying to new recoveries):

$$\mathbb{P}(\#\text{ new infections} \mid I \in \mathcal{B}_b) \quad := \quad \mathbb{P}(\#\text{ new infections} \mid I = \check{I}_b) \tag{29}$$

$$\mathbb{P}(\#\text{ new recoveries} \mid I \in \mathcal{B}_b) \quad := \quad \mathbb{P}(\#\text{ new recoveries} \mid I = \check{I}_b) \ . \tag{30}$$

Ascertainment bias model – assumptions and caveats

Debias-1 Spatial homogeneity of δ across LTLAs within a PHE region. The fact that we see relatively low variation in δ at each time point across PHE regions in Fig. 3, particularly after October 2020, is consistent with a finer-scale spatial homogeneity assumption being reasonable.

Debias-2 We handle prevalence in a reduced-dimension space of bins as described in SI section *Interval-based prevalence inference – set-up and assumptions*

Debias-3 (In)stability of ascertainment mechanism. It is clear from Fig. 3 that the ascertainment effects captured by δ can change rapidly and without obvious cause over time. Contemporaneous randomised surveillance data, such as REACT or ONS CIS, allow estimation of δ. However, when predicting prevalence forward in time beyond availability of randomised surveillance data, we are making the implicit assumption that the ascertainment bias remains stable forwards in time, and such results should therefore be interpreted with caution.

PCR+ to infectious mapping – assumptions and caveats

For full details please see Supplementary Information—*PCR positive to infectious mapping – method details.*

Infectious-1 Pillar 1+2 positive test counts, across a four-week period, are used as an approximation to the true *relative* incidence over that time interval at coarse-scale level (e.g. PHE region).

Infectious-2 The probability (with credible intervals) of testing PCR positive when swabbed d days post infection is taken from Fig. 1A of Hellewell et al. [35].

Infectious-3 The infectious interval for an average individual is defined to span days 1 to 11 post infection, based on Fig. 1A of Ferretti et al. [34].

[3] Bins are equally sized on log scale, with interval edges are defined recursively as $e_0 = 0$, $e_b = \lceil e_{b-1}(1 + \varepsilon_B) \rceil$, and ε_B is a fixed constant giving B intervals.

SIR model – discussion, assumptions and caveats

The illustrative epidemic model we implement here has one of the simplest SIR compartmental structures available, as summarised in Supplementary Information–*SIR model – discussion, assumptions and caveats* and particularly Assumption SIR-2. Other teams have developed more realistic and sophisticated compartmental models of transmission, reflecting for example that individuals are not immediately infectious after being infected [18, 42, 43, 44]. Importantly, these are able to relate epidemiological disease dynamics to outcomes far downstream, such as hospitalisation and deaths. The fact that a large number and variety of models has been developed can be viewed as a strength, as demonstrated by efficacy of ensembles of multi-model forecasts to inform policy on future resource needs and population impacts [27]. One attractive feature of such model ensembles is that their forecasts may be relatively robust to changes in spatiotemporal and compartmental dynamics over the course of an epidemic. Notably, the de-biased prevalence likelihood outputted in Results–*Cross-sectional local prevalence from targeted testing data* is agnostic to the downstream epidemic model, and so there might be benefits to incorporating it into such multi-compartment epidemic models.

SIR-1 The population is homogeneous within an LTLA, with each individual equally likely to be infected

SIR-2 We assume individuals become instantly infectious and recover at a fixed rate $\gamma = 7$ days, i.e. with no spatiotemporal variation, and with recovery time distributed exponentially with mean $1/\gamma$.

SIR-3 Any projections forward in time are made under the implicit assumption that there is no change in NPIs, such as tiering or lockdown status, affecting the LTLA.

SIR-4 We do not include age, ethnicity or deprivation indices in our model, and so epidemiological parameter estimates are to be interpreted as an average across these strata (with unknown weights).

SIR-5 We do not explicitly model transmission between regions or the demographic effects of births, deaths and migration – the SIR model is fitted to each LTLA separately. While it would be possible to account for transmission between LTLAs [45], this dramatically increases the number of parameters to be estimated and consequently the computational burden of the model. Given that the study period here is almost all in lockdown, the effect of transmission between LTLAs is relatively small. In non-lockdown periods, epidemic models allowing for inter-region transmission could be beneficial.

SIR-6 The number of new infections in the stochastic SIR model is modelled as a Poisson approximation, approximating the 'true' Binomial conditional distribution.

Gaussian approximation for δ

We approximate the cross-sectional component of the EB prior for δ using a moment-matched Gaussian approximation (see (12)). Supplementary Fig. 2 illustrates the suitability of this approximation for PHE regions London and the North West across nine weeks.

SIR model details

We implement a DTMC SIR epidemic model based on the standard model as described in ([36], Chapter 3). As we choose Δt to be a day/week, we allow multiple infections and recoveries in a time interval width Δt; this requires derivation of Markov transition probabilities between all states (rather than just neighbouring ones), which we do below having established some notation.

Notation

Parameters are subscripted by timepoint index t (indexing week for the analyses presented, with Δt set to one week):

I_t : number of infectious individuals

R_t^+ : number of immune individuals (with infection- and/or vaccination-acquired immunity)

V_t : total number of vaccinated individuals in region (i.e. with vaccine-acquired immunity)

S_t : number of susceptible individuals ($S_t \equiv M - R_t^+ - I_t$)

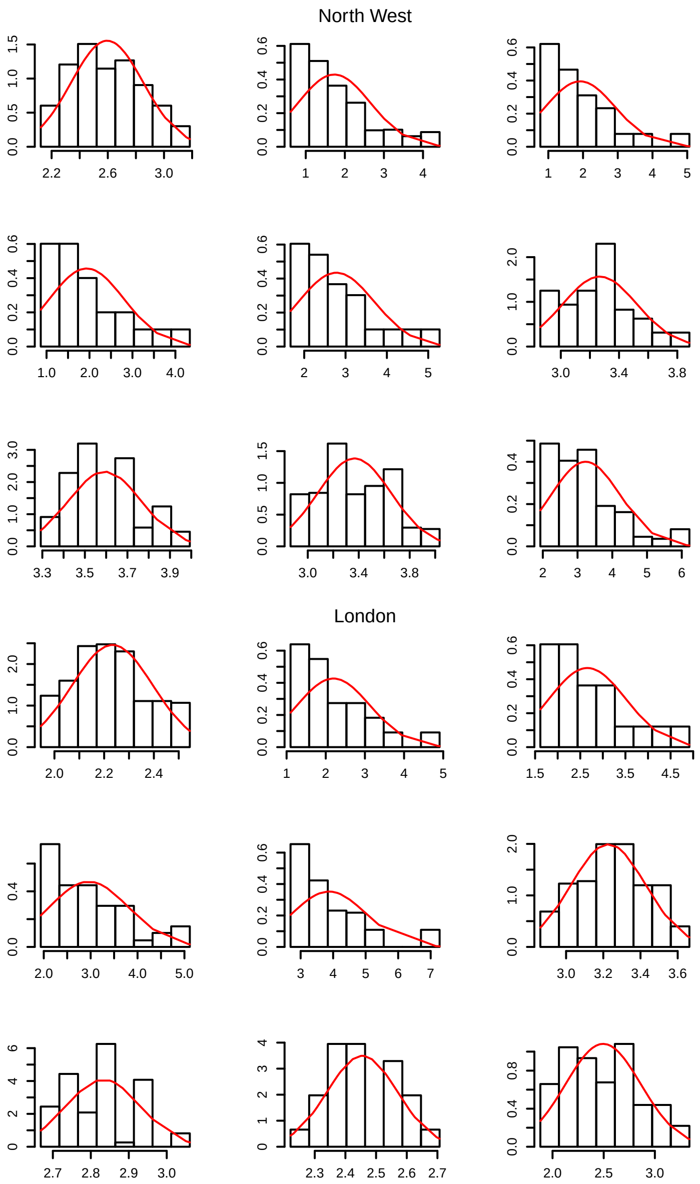

Supplementary Figure 2: Comparison of moment-matched Gaussian EB prior (12) (red lines) with raw estimates (histograms) on δ for PHE regions North West (top) and London (bottom) from 29th November 2020 to 24th January 2021.

ΔQ_t : number of *new* infections in interval $(t-\Delta t,\ t]$

ΔR_t : number of *new* recoveries in interval $(t-\Delta t,\ t]$

ΔV_t : number of vaccinations administered in interval $(t-\Delta t,\ t]$

$\Delta\tilde{V}_t$: number of vaccinations administered to *susceptible* individuals in interval $(t-\Delta t,\ t]$

β_t : transmission rate, i.e. the number of effective contacts in interval $(t-\Delta t,\ t]$

γ : recovery rate, with expected time to recovery $\mathbb{E}[T] = 1/\gamma$

γ_t : probability of recovery in interval $(t-\Delta t,\ t]$, i.e. $\gamma_t := \mathbb{P}(T \le \Delta t)$ where $T \sim \text{Exp}(\gamma)$

$\mathcal{R}^0_t$: basic reproduction number, $\mathcal{R}^0_t \equiv \beta_t/\gamma_t$

$\mathcal{R}_t$: effective reproduction number, $\mathcal{R}_t \equiv \mathcal{R}^0_t S_t/M$

Distribution of the number of new infections ΔQ_t

Under the standard DTMC SIR model, the number of *new* infections, denoted here ΔQ_t, occurring in the time interval Δt up to time t has conditional distribution[4]

$$\mathbb{P}(\Delta Q_t \mid S_{t-1}, \beta_{t-1}, I_{t-1}) = \text{Binomial}\left(\Delta Q_t \mid S_{t-1},\ \frac{\beta_{t-1} I_{t-1}}{M}\right)\ . \tag{31}$$

The probability in (31) can be parameterised by the effective reproduction number, $\mathcal{R}_t$:

$$\mathcal{R}_t := \frac{\beta_t S_t}{\gamma_t M} \tag{32}$$

$$\mathbb{P}(\Delta Q_t \mid S_{t-1}, \mathcal{R}_{t-1}, I_{t-1}) \equiv \text{Binomial}\left(\Delta Q_t \mid S_{t-1},\ \frac{\gamma_t \mathcal{R}_{t-1} I_{t-1}}{S_{t-1}}\right)\ . \tag{33}$$

We approximate (33) with a Poisson distribution as follows [46]:[5]

$$\mathbb{P}(\Delta Q_t \mid \mathcal{R}_{t-1}, I_{t-1}) := \text{Poisson}\left(\Delta Q_t \mid \gamma_t \mathcal{R}_{t-1} I_{t-1}\right)\ . \tag{34}$$

Distribution of the number of new recoveries ΔR_t

The number of *new* recoveries, denoted ΔR_t, occurring in the time interval Δt up to time t is distributed

$$\mathbb{P}(\Delta R_t \mid I_{t-1}) = \text{Binomial}\left(\Delta R_t \mid I_{t-1},\ \gamma_t\right)\ . \tag{35}$$

[4]Based on each of $S_{t-1} \equiv M - R^+_{t-1} - I_{t-1}$ susceptibles at time $t-1$ being infected independently with probability

$$\begin{aligned}
&\mathbb{P}(\text{Susceptible infected} \mid \beta_{t-1} \text{ effective contacts in } (t-\Delta t,\ t])\\
&\quad = 1 - \mathbb{P}(\text{Susceptible is not infected} \mid \beta_{t-1} \text{ effective contacts})\\
&\quad = 1 - \mathbb{P}(\text{A random effective contact is with a noninfectious individual})^{\beta_{t-1}}\\
&\quad = 1 - \left(1 - \frac{I_{t-1}}{M}\right)^{\beta_{t-1}}\\
&\quad = \frac{\beta_{t-1} I_{t-1}}{M} + O\left(\left[\frac{I_{t-1}}{M}\right]^2\right)\ .
\end{aligned}$$

[5]According to Rule 2 in [46], the Poisson approximation is reasonable when both of these inequalities hold:

$$\gamma_t \mathcal{R}_{t-1} I_{t-1} > 5$$
$$\frac{\gamma_t \mathcal{R}_{t-1} I_{t-1}}{S_{t-1}} < \frac{1}{2}\ .$$

Of the two, the first is the least likely to obtain, but is still reasonable under most circumstances. For a simple example, if we set $\gamma_t = 1$ and $\mathcal{R}_{t-1} = 1$, the number of infectious individuals $I_{t-1} > 5$ is sufficient for the approximation to be reasonable.

Transition probabilities for the number of infectious individuals I_t

The change in the number of infectious individuals at time t, ΔI_t can then be expressed as

$$\Delta I_t = \Delta Q_t - \Delta R_t$$

this and so the conditional distribution for ΔI_t follows from (34) and (35):

$$\mathbb{P}(\Delta I_t \mid I_{t-1}, \mathcal{R}_{t-1}) = \sum_{\Delta R_t=0}^{I_{t-1}} \Big\{ \text{Binomial}(\Delta R_t \mid I_{t-1},\ \gamma_t) \times \text{Poisson}\left(\Delta I_t + \Delta R_t \mid \gamma_t \mathcal{R}_{t-1} I_{t-1}\right) \Big\} . \quad (36)$$

Interval-to-interval transition probabilities are evaluated as

$$\begin{aligned} \mathbb{P}(I_t \in \mathcal{B}_{b'} \mid I_{t-1} \in \mathcal{B}_b, \mathcal{R}_{t-1}) &= \sum_{k \in \mathcal{B}_b} \mathbb{P}(I_{t-1} = k \mid I_{t-1} \in \mathcal{B}_b) \times \mathbb{P}(k + \Delta I_t \in \mathcal{B}_{b'} \mid I_{t-1} = k, \mathcal{R}_{t-1}) \\ &= \sum_{k \in \mathcal{B}_b} \frac{1}{e_b - e_{b-1}} \times \mathbb{P}(k + \Delta I_t \in \mathcal{B}_{b'} \mid I_{t-1} = \check{I}_b, \mathcal{R}_{t-1}) \quad (37) \end{aligned}$$

where the first term in the sum at (37) follows from Assumption 2 at (28), and the second term is conditional on prevalence at bin midpoint ($I_{t-1} = \check{I}_b$) based on Assumption 3 at (29)-(30), and can be evaluated using (36).

Transition probabilities for the number of immune individuals R_t^+

Denote by ΔV_t the number of vaccinations administered in interval $(t - \Delta t,\ t]$. Only a subgroup of those individuals vaccinated at time t may have been susceptible at time $t - \Delta t$; we denote the number in the subgroup by $\Delta \tilde{V}_t$ ($\leq \Delta V_t$), and evaluate its conditional distribution as follows:

$$\Delta \tilde{V}_t := \#\ \text{susceptibles newly vaccinated in}\ (t - \Delta t, t]$$

$$\mathbb{P}(\Delta \tilde{V}_t \mid \Delta V_t, R_{t-1}^+, I_{t-1}) = \text{HyperGeom}(\Delta \tilde{V}_t \mid M - V_t,\ M - R_{t-1}^+ - I_{t-1},\ \Delta V_t)\ , \quad (38)$$

where V_t is the current number of vaccinated individuals in the population (with $\Delta V_t \equiv V_t - V_{t-1}$). The total number of immune, i.e. vaccinated and/or recovered, individuals at time t (denoted R_t^+) can then be represented by the recurrence

$$R_t^+ = R_{t-1}^+ + \Delta R_t + \Delta \tilde{V}_t\ .$$

This leads to the Markov conditional distribution for R_t^+ via convolution of (35) with (38)

$$\mathbb{P}(R_t^+ \mid R_{t-1}^+, I_{t-1}, \Delta V_t) = \sum_{\Delta R_t=0}^{I_{t-1}} \Big\{ \text{Binomial}\left(\Delta R_t \mid I_{t-1},\ \gamma_t\right) \times \text{HyperGeom}(R_t^+ - R_{t-1}^+ - \Delta R_t \mid M - V_{t-1},\ M - R_{t-1}^+ - I_{t-1},\ \Delta V_t) \Big\} . \quad (39)$$

The above treatment of immunity assumes individuals are made permanently immune immediately through either vaccination or infection. It would be straightforward to relax the above formulation to allow for more sophisticated treatment of immunity, for example specifying (a) a delay in vaccine effects, (b) incomplete vaccine efficacy (e.g. in the case of novel variants), or (c) decaying immunity over time.

Inference on the basic reproduction number

The basic reproduction number at time t, $\mathcal{R}_t^0$ is related to the effective reproduction number $\mathcal{R}_t$ by the following equation,

$$\mathcal{R}_t^0 = \frac{S_t}{M} \mathcal{R}_t, \quad (40)$$

where M is the total number of individuals and S_t is the number of susceptible individuals at time t. Recall that $S_t \equiv M - R_t^+ - I_t$ where R_t^+ is the number of immune individuals and I_t is the number of infectious individuals, both of which are estimated by our DTMC SIR model. We can plug in these estimates into (40) to estimate $\mathcal{R}_t^0$ for a given LTLA. Supplementary Fig. 4 plots $\mathcal{R}_t^0$ and $\mathcal{R}_t$ for a selection of LTLAs.

PCR positive to infectious mapping – method details

Recall we require $\mathbb{P}(\tilde{I} \mid I)$ in (3), which is the probability distribution on the number of PCR positive individuals $\tilde{I}$ given the number of infectious individuals I. This can be expressed via Bayes' theorem as

$$\mathbb{P}(\tilde{I} \mid I) \propto \mathbb{P}(I \mid \tilde{I})\mathbb{P}(\tilde{I}) \tag{41}$$

where the likelihood is binomial:

$$\mathbb{P}(I \mid \tilde{I}) = \text{Binomial}(I \mid \tilde{I},\ \mathbb{P}(\text{Infectious} \mid \text{PCR positive}))\ . \tag{42}$$

To target the $\mathbb{P}(\text{Infectious} \mid \text{PCR positive})$ success probability in (42), we introduce the following notation:

$$\text{Infected}_t \equiv \text{Individual } \textbf{becomes} \text{ infected in week } t \tag{43}$$
$$\text{Infectious}_t \equiv \text{Individual is infectious in week } t \tag{44}$$
$$\text{PCR}+_t \equiv \text{Individual is PCR positive from swab taken in week } t \tag{45}$$

and proceed as follows:[6]

$$\mathbb{P}(\text{Infectious}_t \mid \text{PCR}+_t) \tag{46}$$
$$= \frac{\mathbb{P}(\text{Infectious}_t \wedge \text{PCR}+_t)}{\mathbb{P}(\text{PCR}+_t)} \tag{47}$$
$$= \frac{\sum_{k=0}^{3} \mathbb{P}(\text{Infectious}_t \wedge \text{PCR}+_t \mid \text{Infected}_{t-k})\mathbb{P}(\text{Infected}_{t-k})}{\sum_{k=0}^{3} \mathbb{P}(\text{PCR}+_t \mid \text{Infected}_{t-k})\mathbb{P}(\text{Infected}_{t-k})} \tag{48}$$
$$= \frac{\sum_{k=0}^{3} \mathbb{P}(\text{Infectious}_t \mid \text{Infected}_{t-k})\mathbb{P}(\text{PCR}+_t \mid \text{Infected}_{t-k})\mathbb{P}(\text{Infected}_{t-k})}{\sum_{k=0}^{3} \mathbb{P}(\text{PCR}+_t \mid \text{Infected}_{t-k})\mathbb{P}(\text{Infected}_{t-k})}\ , \tag{49}$$

where, at (49), we assumed conditional independence between Infectious_t and $\text{PCR}+_t$ conditional on Infected_{t-k}. Also, at (48), we assumed that testing PCR positive implies that an individual was infected at most four weeks prior to being swabbed, which is consistent with Fig. 1A of [35] (data input 2 below). We import three distinct data inputs to estimate the various terms in (49).

Data input 1 – Infectious interval

Fig. 1A of Ferretti et al. [34] shows the estimated probability density function of the serial interval for SARS-CoV-2 transmission – we denote this density function $f_{\text{Fer}}(d)$. Noting the support of this density to be approximately $[1, 11]$, we specify that an average individual is infectious between days 1 to 11. Formally we define, independently for each individual in the population,

$$\begin{aligned} &\mathbb{P}(\text{Infectious on } d\text{th day post-infection}) := \\ &\quad \mathbb{I}\left\{\mathbb{E}_X[\mathbb{P}(\text{individual } X \text{ Infectious on } d\text{th day post-infection})] > 0\right\} \\ &\quad \approx \begin{cases} 1 & \text{if } f_{\text{Fer}}(d) > 0, \text{ i.e. if } 1 \le d \le 11 \\ 0 & \text{otherwise} \end{cases} \end{aligned}$$

where X denotes an individual selected uniformly at random from the population. We can use this to estimate the $\mathbb{P}(\text{Infectious}_t \mid \text{Infected}_{t-k})$ term appearing in the numerator of (49) as follows

$$\mathbb{P}(\text{Infectious}_t \mid \text{Infected}_{t-k}) \approx \begin{cases} 6/7 & k = 0 \\ 5/7 & k = 1 \\ 0 & k > 1\ . \end{cases} \tag{50}$$

Data input 2 – PCR positive interval

Fig. 1A of Hellewell et al. [35] plots posterior probabilities (with credible intervals) of testing PCR positive when swabbed d days post infection. We denote this data input

$$\mathbb{P}_{\text{Hel}}(\text{PCR}+ \mid \text{swabbed day } d \text{ after becoming infected}) \tag{51}$$

[6] We use $\wedge$ to denote logical AND.

and use it to estimate the term $\mathbb{P}(\text{PCR}+_t \mid \text{Infected}_{t-k})$ appearing twice in (49), evaluating the following estimator for each $k = 0, \ldots, 3$:

$$\mathbb{P}(\text{PCR}+_t \mid \text{Infected}_{t-k}) \approx \frac{1}{7} \sum_{d=7k}^{7(k+1)-1} \mathbb{P}_{\text{Hel}}(\text{PCR}+ \mid \text{swabbed day } d \text{ after becoming infected}) \tag{52}$$

Hellewell et al. [35] helpfully provide reproducible scripts[7] and we use these to extract the posterior distribution on $\mathbb{P}_{\text{Hel}}(\text{PCR}+ \mid \text{swabbed day } d \text{ after becoming infected})$ from their Fig. 1A, whose uncertainty we propagate to estimator (52) and onwards to (49), yielding a distribution on $\mathbb{P}(\text{Infectious}_t \mid \text{PCR}+_t)$ which we take forward approximated by a moment-matched Beta distribution (at each week t) to be used as an EB conjugate prior on the success probability in (42).

Data input 3 – Pillar 1+2 incidence

For the purposes of adjusting the PCR positive map to changing incidence, we use the raw regional weekly positive test counts $n_{0:T}$, where we denote weeks by $t = 0, \ldots, T$. We use this data input to estimate the term $\mathbb{P}(\text{Infected}_{t-k})$ appearing twice in (49), evaluating the following estimator for each $k = 0, \ldots, 3$:[8]

$$\begin{aligned}
\mathbb{P}(\text{Infected}_{t-k}) &= \mathbb{P}(\text{Infected}_{t-k} \wedge \left[\vee_{k'=0}^{3} \text{Infected}_{t-k'}\right]) && (53)\\
&= \mathbb{P}(\text{Infected}_{t-k} \mid \vee_{k'=0}^{3} \text{Infected}_{t-k'}) \mathbb{P}(\vee_{k'=0}^{3} \text{Infected}_{t-k'}) && (54)\\
&\approx \frac{n_{t-k}}{\sum_{k'=0}^{3} n_{t-k'}} \mathbb{P}(\vee_{k'=0}^{3} \text{Infected}_{t-k'}) && (55)
\end{aligned}$$

which can be directly substituted for $\mathbb{P}(\text{Infected}_{t-k})$ in top and bottom of (49) with the second term on the right of (55) cancelling between numerator and denominator, and therefore not requiring evaluation. We note that we are using raw counts to model relative incidence over a relatively short period (four weeks), which is making the assumption that the bias is relatively stable over this timeframe (see Assumption Infectious-1 in SI–*PCR+ to infectious mapping – assumptions and caveats*).

Estimating antigen testing false positive rate – method details

We estimate the type I error rate α for PCR antigen testing based on REACT data under the following model:

$$\mathbb{P}(u_{J,t} \text{ of } U_{J,t} \mid \pi_{J,t}, \alpha) = \text{Binomial}(u_{J,t} \mid U_{J,t},\ \alpha + \pi_{J,t}) \tag{56}$$

where $u_{J,t}$ and $U_{J,t}$ are positive and total REACT test counts in PHE region J for week t, and $\pi_{J,t}$ denotes the corresponding prevalence proportion (the proportion of individuals in region J for week t who would test PCR positive if tested). For inference we define the following i.i.d. empirical Bayes prior for the $\pi_{J,t}$:

$$p(\pi_{J,t}) = \hat{F}(\pi_{J,t}) \tag{57}$$

where $\hat{F}$ denotes the empirical CDF of the prevalence proportion estimates, i.e. of $\{u_{J,t}/U_{J,t} : J = 1, \ldots, 9;\ t = 1, \ldots, T\}$. We then evaluate the following marginal log likelihood:

$$\log p(\alpha) = \sum_{J,t} \log \int_0^1 \mathbb{P}(u_{J,t} \text{ of } U_{J,t} \mid \pi_{J,t}, \alpha) \hat{F}(\pi_{J,t}) d\pi_{J,t}\ . \tag{58}$$

Supplementary Fig. 5 plots $\log p(\alpha)$; we select the value $\alpha = 0.0003$, rounding the maximum likelihood estimate to one significant figure.

Supplementary results

Sensitivity analyses

Prior hyperparameters for δ

The EB prior for δ depends on two hyperparameters: σ_ϵ controls the variance of the white noise associated with each individual time point, while ψ controls the degree of autocorrelation from one time point to the next. Supplementary Fig. 6 shows the estimates for prevalence and $\mathcal{R}_t$ of infectious individuals using different values of these two hyperparameters. Note that in the main text, we present results using $\sigma_\epsilon = 1$ and $\psi = 0.99$.

[7] https://github.com/cmmid/pcr-profile

[8] We use $\vee$ to denote logical OR.

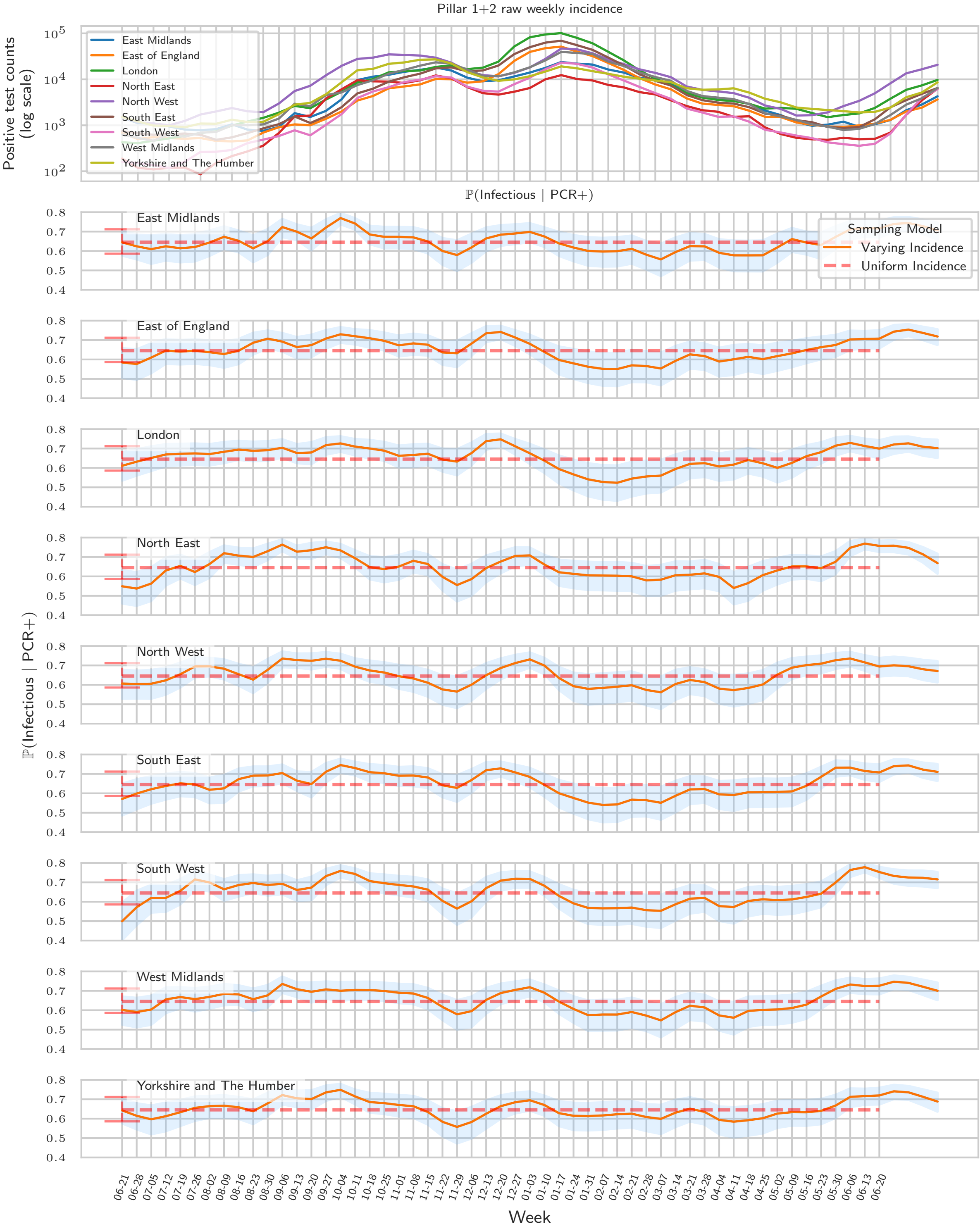

Supplementary Figure 3: EB prior on ℙ(Infectious | PCR positive) by week and PHE region. The top panel shows raw weekly Pillar 1+2 incidence for the nine PHE regions; this is to provide intuition for the Varying incidence model in the panels below. The bottom nine panels display the prior we place on $\mathbb{P}(\text{Infectious}_t \mid \text{PCR+}_t)$, which is specific to week and region for the Varying incidence model, but is constant across weeks/regions for the Uniform incidence model (see legend in panel 2). Error bars (at left of panel for Uniform incidence; around curve for Varying incidence) represent 95% credible intervals.

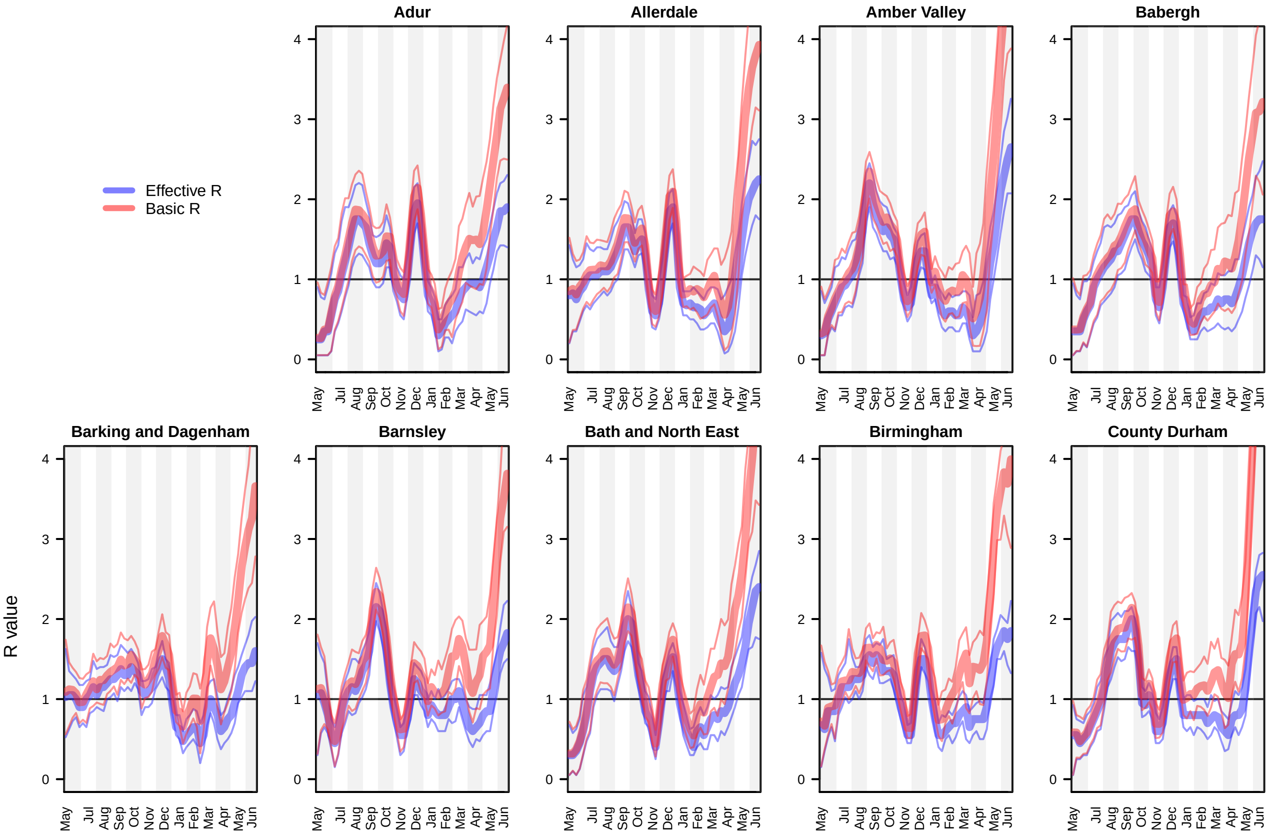

Supplementary Figure 4: Comparison of $\mathcal{R}_t^0$ and $\mathcal{R}_t$ estimates.

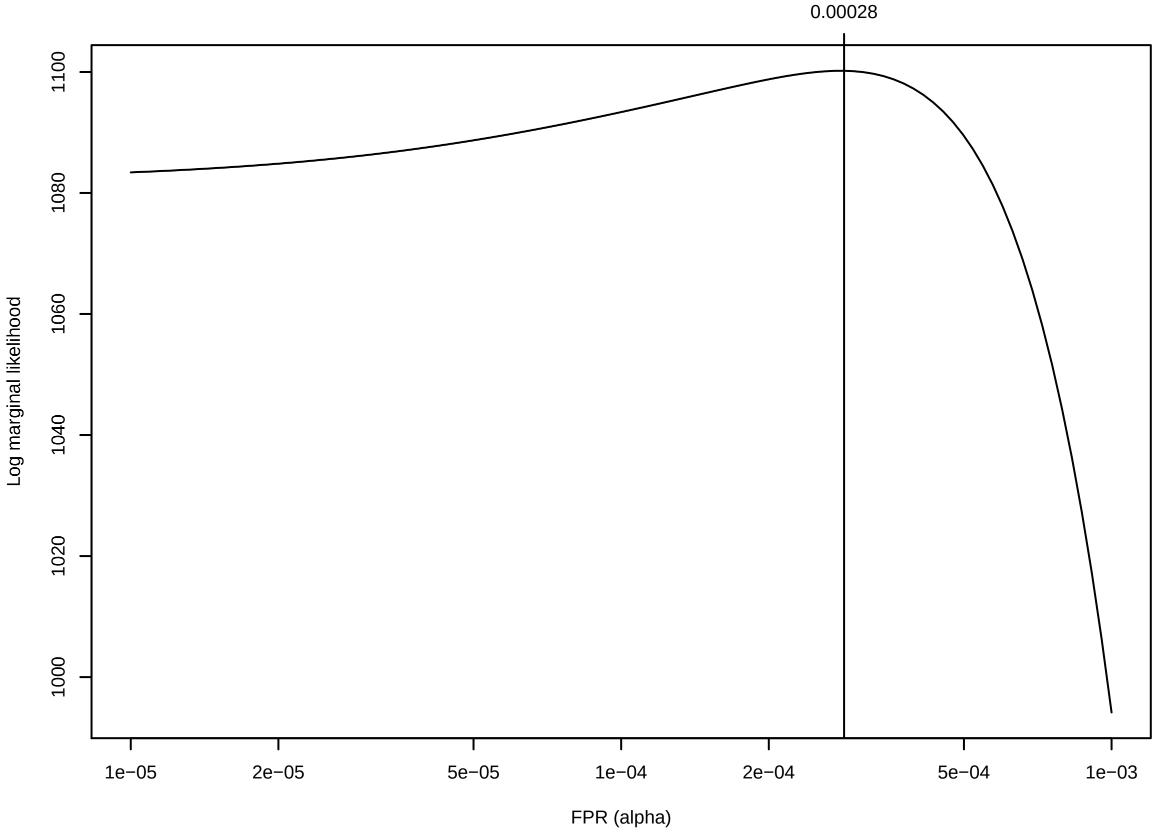

Supplementary Figure 5: Estimated log marginal likelihood for false positive rate of PCR swab testing. Details are given in SI—*Estimating antigen testing false positive rate – method details*, with the log marginal likelihood evaluated as described at (58).

Sensitivity and specificity of PCR tests

PCR tests are not perfect and are subject to both false positives and false negatives. In our analysis, we account for imperfect testing via the false positive rate, α, and the false negative rate, β (see (8)). Supplementary Fig. 7 shows the estimates for prevalence and $\mathcal{R}_t$ of infectious individuals using different values of these two hyperparameters. Note that in the main text, we present results using $\alpha = 3 \times 10^{-4}$ and $\beta = 0.05$.

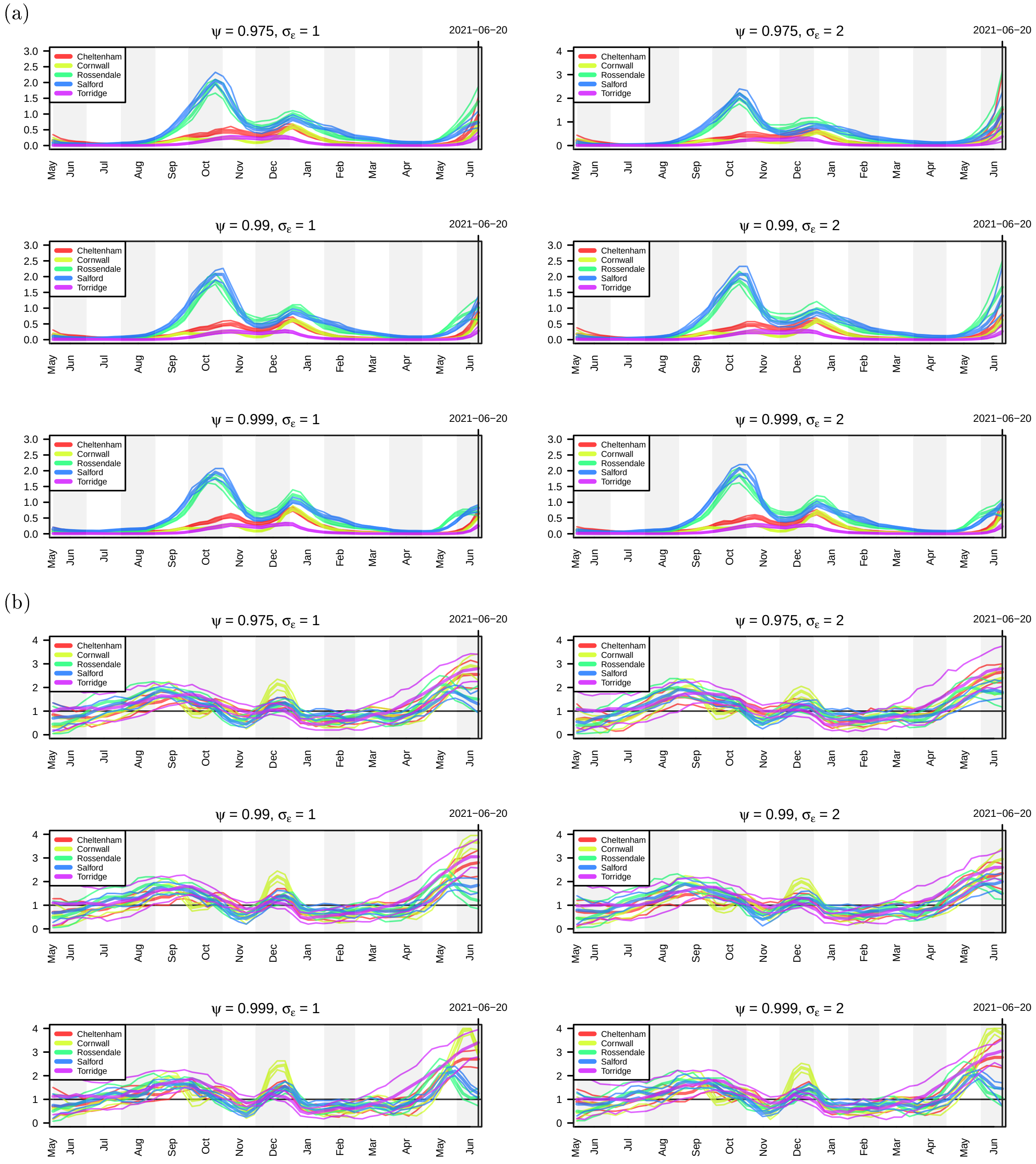

Supplementary Figure 6: (a) Estimates of prevalence of infectious individuals for five LTLAs using different values of the hyperparameters σ_ϵ and ψ controlling the smoothness of the bias parameter δ. (b) Estimates of prevalence of infectious individuals five LTLAs using different values of the false positive rate α and false negative rate β.

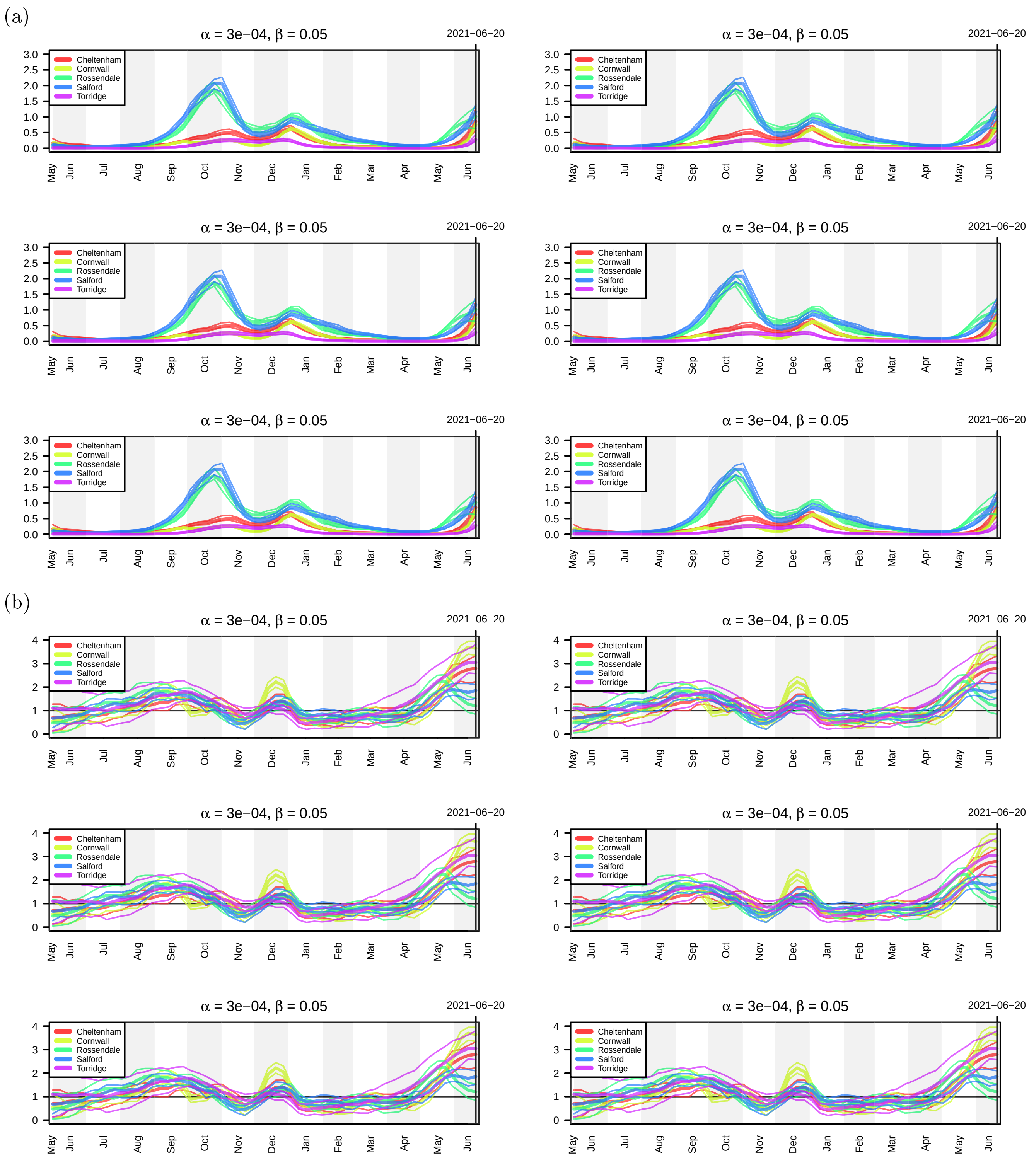

Supplementary Figure 7: (a) Estimates of $\mathcal{R}_t$ of infectious individuals five LTLAs using different values of the false positive rate α and false negative rate β controlling the smoothness of the bias parameter δ. (b) Estimates of prevalence of infectious individuals for five LTLAs using different values of the hyperparameters σ_ϵ and ψ controlling the smoothness of the bias parameter δ.

References

[40] Department of Health and Social Care. Lateral flow device specificity in phase 4 (post-marketing) surveillance. https://www.gov.uk/government/publications/lateral-flow-device-specificity-in-phase-4-post-marketing-surveillance.

[41] Office of National Statistics. Coronavirus (covid-19) infection survey: antibody data for the uk, january 2021. https://www.ons.gov.uk/peoplepopulationandcommunity/healthandsocialcare/conditionsanddiseases/articles/coronaviruscovid19infectionsinthecommunityinengland/antibodydatafortheukjanuary2021.

[42] Overton, C. E. et al. Using statistics and mathematical modelling to understand infectious disease outbreaks: COVID-19 as an example. Infectious Disease Modelling **5**, 409–441 (2020). URL https://www.sciencedirect.com/science/article/pii/S2468042720300245.

[43] Keeling, M. J. et al. Predictions of COVID-19 dynamics in the UK: Short-term forecasting and analysis of potential exit strategies. PLOS Computational Biology **17**, e1008619 (2021). URL https://journals.plos.org/ploscompbiol/article?id=10.1371/journal.pcbi.1008619. Publisher: Public Library of Science.

[44] Keeling, M. J. et al. Fitting to the UK COVID-19 outbreak, short-term forecasts and estimating the reproductive number. medRxiv 2020.08.04.20163782 (2020). URL http://medrxiv.org/content/early/2020/09/29/2020.08.04.20163782.abstract.

[45] Brown, G. D., Porter, A. T., Oleson, J. J. & Hinman, J. A. Approximate Bayesian computation for spatial SEIR(S) epidemic models. Spatial and Spatio-Temporal Epidemiology **24**, 27–37 (2018).

[46] Schader, M. & Schmid, F. Two Rules of Thumb for the Approximation of the Binomial Distribution by the Normal Distribution. The American Statistician (2012). URL https://www.tandfonline.com/doi/abs/10.1080/00031305.1989.10475601. Publisher: Taylor & Francis Group.
