# Supplementary material for: Improving local prevalence estimates of SARS-CoV-2 infections using a causal debiasing framework
Source: Nat Microbiol. 2021 Dec 31;7(1):97–107. doi: 10.1038/s41564-021-01029-0 (PMC8727294; doi:10.1038/s41564-021-01029-0)
Supplement: Supplementary file 2 — Reporting Summary [file 41564_2021_1029_MOESM2_ESM.pdf]

nature portfolio

Corresponding author(s): Prof. Chris Holmes

Last updated by author(s): Nov 11, 2021

Reporting Summary

Nature Portfolio wishes to improve the reproducibility of the work that we publish. This form provides structure for consistency and transparency in reporting. For further information on Nature Portfolio policies, see our Editorial Policies and the Editorial Policy Checklist.

Statistics

For all statistical analyses, confirm that the following items are present in the figure legend, table legend, main text, or Methods section.

n/a	Confirmed	
☐	☒	The exact sample size (*n*) for each experimental group/condition, given as a discrete number and unit of measurement
☐	☒	A statement on whether measurements were taken from distinct samples or whether the same sample was measured repeatedly
☐	☒	The statistical test(s) used AND whether they are one- or two-sided<br>*Only common tests should be described solely by name; describe more complex techniques in the Methods section.*
☐	☒	A description of all covariates tested
☐	☒	A description of any assumptions or corrections, such as tests of normality and adjustment for multiple comparisons
☐	☒	A full description of the statistical parameters including central tendency (e.g. means) or other basic estimates (e.g. regression coefficient) AND variation (e.g. standard deviation) or associated estimates of uncertainty (e.g. confidence intervals)
☐	☒	For null hypothesis testing, the test statistic (e.g. *F*, *t*, *r*) with confidence intervals, effect sizes, degrees of freedom and *P* value noted<br>*Give P values as exact values whenever suitable.*
☐	☒	For Bayesian analysis, information on the choice of priors and Markov chain Monte Carlo settings
☐	☒	For hierarchical and complex designs, identification of the appropriate level for tests and full reporting of outcomes
☒	☐	Estimates of effect sizes (e.g. Cohen's *d*, Pearson's *r*), indicating how they were calculated

Our web collection on statistics for biologists contains articles on many of the points above.

Software and code

Policy information about availability of computer code

Data collection	The R script used to download the data from public repositories prior to analysis in this manuscript is available at: https://github.com/alan-turing-institute/jbc-turing-rss-testdebiasing/scripts/00_download_data.R
Data analysis	The R scripts used to generate the results in this manuscript are available at: https://github.com/alan-turing-institute/jbc-turing-rss-testdebiasing

For manuscripts utilizing custom algorithms or software that are central to the research but not yet described in published literature, software must be made available to editors and reviewers. We strongly encourage code deposition in a community repository (e.g. GitHub). See the Nature Portfolio guidelines for submitting code & software for further information.

Data

Policy information about availability of data

All manuscripts must include a data availability statement. This statement should provide the following information, where applicable:

- Accession codes, unique identifiers, or web links for publicly available datasets
- A description of any restrictions on data availability
- For clinical datasets or third party data, please ensure that the statement adheres to our policy

With the exception of the Alpha VOC 202012/01 analysis, all data underlying the results presented here are publicly available. Randomised surveillance data comes from the REACT study (data downloaded from https://github.com/mrc-ide/reactidd/tree/master/inst/extdata). From REACT, we aggregate weekly test counts at the spatially coarse-scale level (PHE region) and, for validation purposes but not model fitting, use round-aggregated counts at the fine-scale level (LTLA), for rounds 7 to 11. The combined weekly Pillar 1 and Pillar 2 data are publicly available for download (https://www.gov.uk/government/publications/nhs-test-and-trace-england-statistics-14-january-to-20-january-2021). Note that lateral flow test results are not included in the these weekly summaries. We downloaded Rt estimates

outputted by the Imperial College team's Epidemia model from https://imperialcollegelondon.github.io/covid19local/downloads/UK_hotspot_Rt_estimates.csv on 2021-10-13, and we provide a copy of that downloaded file in our Zenodo repository at <URL to be provided>.

Field-specific reporting

Please select the one below that is the best fit for your research. If you are not sure, read the appropriate sections before making your selection.

☐ Life sciences ☒ Behavioural & social sciences ☐ Ecological, evolutionary & environmental sciences

For a reference copy of the document with all sections, see nature.com/documents/nr-reporting-summary-flat.pdf

Behavioural & social sciences study design

All studies must disclose on these points even when the disclosure is negative.

Study description

Methods for studying COVID-19 epidemiology by analysing community testing data (quantitative count data, comprising the number positive and total number tested)

Research sample

We collectively analyse two types of community testing data: The REal-time Assessment of Community Transmission (REACT) study is a nationally representative prevalence survey of SARS-CoV-2 based on repeated cross-sectional samples from a representative subpopulation defined via (stratified) random sampling from England's National Health Service patient register. Pillar 1 and Pillar 2 PCR test data form the main part of the UK government's national antigen testing strategy. Pillar 1 tests refer to "all swab tests performed in Public Health England (PHE) labs and National Health Service (NHS) hospitals for those with a clinical need, and health and care workers", and Pillar 2 comprises "swab testing for the wider population". The Pillar 1+2 research sample is dynamic, and is not representative of the population as a whole. In particular, Pillar 1+2 is enriched for NHS workers, individuals with symptoms, and those who self-select the testing, all of which can affect the demographic representation in the targeted Pillar 1+2 individuals. The REACT research sample is however designed to be nationally representative by stratified random sampling. We are able to use these REACT data to account for the ascertainment bias implicit in Pillar 1+2 data.

For Pillar 1+2, the age and sex demographic breakdowns for the number of tests conducted are available here: https://assets.publishing.service.gov.uk/government/uploads/system/uploads/attachment_data/file/1007158/Demographic_LA_tables_Week_60.ods As an example, for the week commencing 2021-01-07, the age breakdown was as follows: 0-9yr: 83,864 (4%); 10-19yr: 127,482 (6%); 20-29yr: 394,029 (17%); 30-39yr: 406,058 (18%); 40-49yr: 364,772 (16%); 50-59yr: 397,950 (18%); 60-69yr: 223,665 (10%); 70-79yr: 119,226 (5%); 80-89yr: 93,704 (4%); 90+yr: 49,908 (2%). The sex split was Males: 886,970 (39%); Females: 1,373,688 (61%).

For REACT, the age-stratified breakdown for round 8 is available here https://github.com/mrc-ide/reactidd/blob/master/inst/extdata/region_age_week_aggregated/round_8_go.csv. As an example, for REACT round 8, spanning 6th-22nd Jan 2021, the age breakdown is 5-12yr: 11,545 (7%); 13-17yr: 8,842 (5%); 18-24yr: 6,614 (4%); 25-34yr: 14,715 (9%); 35-44yr: 21,357 (13%); 45-54yr: 27,583 (17%); 55-64yr: 31,665 (19%); 65+yr: 43,246 (26%). Sex split metadata for REACT study are not publicly available.

The rationale for choosing Pillar 1+2 positive and total counts is that these data are the most highly publicised case counts in England and they are regularly made publicly available, which allows ready reproducibility and ongoing implementation of our methodology. The rationale for choosing the REACT study is that we require randomised surveillance data in order to correct for ascertainment bias. REACT is one of the two major randomised surveillance studies, along with the office for National statistics COVID-19 infection survey, ONS CIS. We specifically use REACT data here because they are regularly made publicly available in raw form (positive and total counts) at the PHE region level, which is compatible with our downstream statistical modelling, and which allows transparency and reproducibility of our findings.

Sampling strategy

For the REACT data, participants were included in the tested group through stratified random sampling. For the Pillar 1+2 data, however, there is strong ascertainment bias, since infected individuals are more likely to be chosen for testing (e.g. frontline workers, symptomatics). In our paper we correct for this ascertainment bias in the Pillar 1+2 data by designing a causal model and thereby adjusting for the bias to obtain accurate estimates of local prevalence. We have used two datasets in our study: Pillar 1+2 and REACT. These have the advantage of being both publicly available (allowing reproducibility of our results), and we demonstrate in the paper that they are sufficient for us to develop, illustrate and, importantly, to validate our methodology (See section "Accuracy validation using ultra-coarse and incomplete data to estimate delta")

Data collection

In the REACT study, participants self-gathered throat and nose swab samples; no researcher was typically present during sample collection and other members of the public could have been present; participants were not blinded to the study hypothesis. Samples were then sent by post to a pre-specified laboratory for processing.

For the Pillar 1+2 data, throat and nose swabs were gathered in various ways. For home testing, participants self-gathered throat and nose swab samples; no researcher was typically present during sample collection and other members of the public could have been present; participants were not blinded to the study hypothesis. In the case of regional or local test sites, or mobile testing units, swabs were gathered by a trained healthcare professional while the participant was seated in a motor vehicle; usually only the researcher and participant were present; neither the researcher nor the participant were blinded to the study hypothesis.

Timing

Between 31st May 2020 and 20th June 2021

Data exclusions

No data were excluded from the analysis

Non-participation	Over the course of the REACT study, there has been a considerable number of individuals who (i) are invited to participate but decline; or (ii) dropped out at some point during the study. We do not have access to these data.
Randomization	For the REACT data, participants were included in the tested group through stratified random sampling.<br><br>For the Pillar 1+2 data there is a strong ascertainment effect since infected individuals are more likely to be chosen for testing (e.g. frontline workers, COVID-19 symptomatic individuals). In our paper we correct for this ascertainment bias in the Pillar 1+2 data by specifying a causal model to adjust for the bias and to obtain accurate estimates of local prevalence. We did not directly control for confounding covariates in our analysis of the Pillar 1+2 data; instead, we indirectly controlled for any potential confounders by estimating the marginal causal probabilities of being tested in the infected and non-infected groups (see Figure 1(a), in which socio-economic status is an example confounder).

Reporting for specific materials, systems and methods

We require information from authors about some types of materials, experimental systems and methods used in many studies. Here, indicate whether each material, system or method listed is relevant to your study. If you are not sure if a list item applies to your research, read the appropriate section before selecting a response.

Materials & experimental systems

n/a	Involved in the study
☒	☐ Antibodies
☒	☐ Eukaryotic cell lines
☒	☐ Palaeontology and archaeology
☒	☐ Animals and other organisms
☐	☒ Human research participants
☒	☐ Clinical data
☒	☐ Dual use research of concern

Methods

n/a	Involved in the study
☒	☐ ChIP-seq
☒	☐ Flow cytometry
☒	☐ MRI-based neuroimaging

Human research participants

Policy information about studies involving human research participants

Population characteristics	See above
Recruitment	The REACT study approached a random sample of the population in England aged five years and above, using the National Health Service (NHS) records and invited them to join the study. There is some residual degree of non-response bias in the REACT study despite it being a randomised study; we illustrate this residual ascertainment effect in Figure 1(a) with a dashed blue arrow denoting non-response bias associated with socioeconomic status (SES). If metadata on SES were available, then this bias could be mitigated; we did not have access to SES data, and the likely impact on our results is that the debiased prevalence is mildly unduly weighted towards those strata in the population which are more likely to respond to REACT's invitation to participate.<br><br>Individuals tested in the Pillar 1+2 data were recruited, self-selected or selected according to place of work, according to a number of potential criteria (e.g. NHS workers, those with COVID-19 symptoms); i.e. the Pillar 1+2 data harbour self-selection and other biases -- it is the purpose of the current work to adjust collectively for these biases.
Ethics oversight	The Alan Turing Institute Ethics Advisory Group

Note that full information on the approval of the study protocol must also be provided in the manuscript.
